# Cell Membrane Coating Technology: A Promising Strategy for Biomedical Applications

**DOI:** 10.1007/s40820-019-0330-9

**Published:** 2019-11-16

**Authors:** Yao Liu, Jingshan Luo, Xiaojia Chen, Wei Liu, Tongkai Chen

**Affiliations:** 1 Science and Technology Innovation Center, Guangzhou University of Chinese Medicine, Guangzhou, 510405 People’s Republic of China; 2 Institute of Clinical Pharmacology, Guangzhou University of Chinese Medicine, Guangzhou, 510405 People’s Republic of China; 3 State Key Laboratory of Quality Research in Chinese Medicine, Institute of Chinese Medical Sciences, University of Macau, Macau, People’s Republic of China; 4 Key Laboratory of Artificial Micro- and Nano-Structures of Ministry of Education, School of Physics and Technology, Wuhan University, Wuhan, 430072 People’s Republic of China

**Keywords:** Cell membrane, Biomimetic nanoparticles, Cancer therapy, Immune modulation, Detoxification

## Abstract

Cell membrane coating technology is an approach to the biomimetic replication of cell membrane properties, and is an active area of ongoing research readily applicable to nanoscale biomedicine. Nanoparticles (NPs) coated with cell membranes offer an opportunity to unite natural cell membrane properties with those of the artificial inner core material. The coated NPs not only increase their biocompatibility but also achieve effective and extended circulation in vivo, allowing for the execution of targeted functions. Although cell membrane-coated NPs offer clear advantages, much work remains before they can be applied in clinical practice. In this review, we first provide a comprehensive overview of the theory of cell membrane coating technology, followed by a summary of the existing preparation and characterization techniques. Next, we focus on the functions and applications of various cell membrane types. In addition, we collate model drugs used in cell membrane coating technology, and review the patent applications related to this technology from the past 10 years. Finally, we survey future challenges and trends pertaining to this technology in an effort to provide a comprehensive overview of the future development of cell membrane coating technology.

## Highlights

- The recent progress on using cell membrane-coated nanoparticles for drug delivery, cancer treatment, vascular disease, immune modulation, and detoxification are summarized in this review.
- The patent applications related to the cell membrane coating technology from the past 10 years are collected, the future challenges and trends pertaining to this technology are comprehensively discussed.
- Unique properties of cell membrane-coated nanoparticles make it a promising strategy for biomedical applications and will make outstanding contributions to human health.

## Graphical abstract

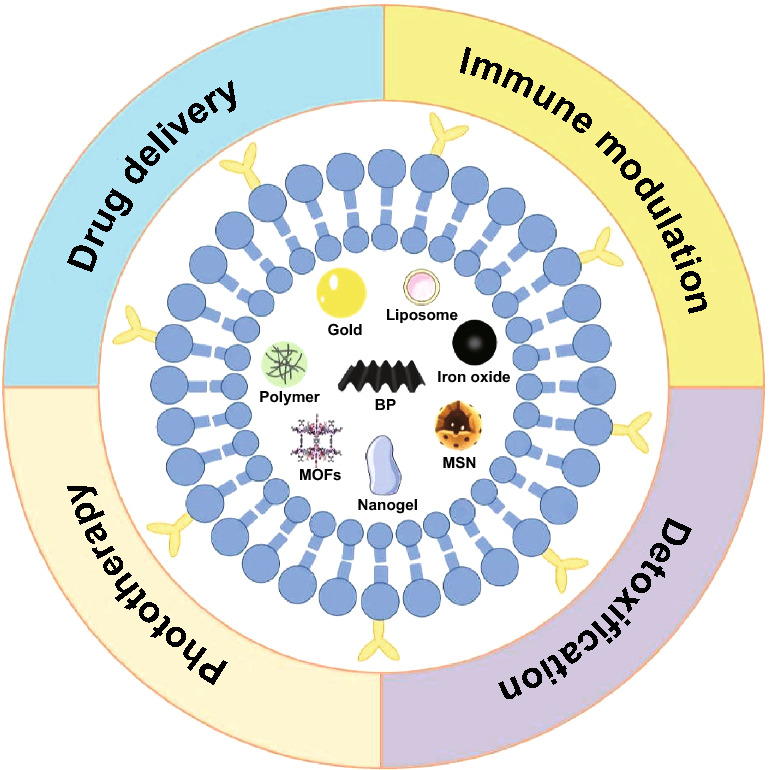

## Introduction

Nanoparticles (NPs) have been extensively explored in diagnostic and therapeutic contexts, with potential applications to drug delivery, photothermal therapy, diagnostic imaging, photodynamic therapy, nucleic acid delivery, and implantable devices [1–6]. NPs offer some advantages: (1) protecting their cargo from inactivation or degradation before target delivery in vivo [7], (2) improving targeting by modifying ligands [8], (3) controlling drug release by changing the composition of NP polymers [9], (4) allowing for batch productions [10]. Despite these advantages, only a few NPs have been assessed in clinical trials and successfully approved by the US Food and Drug Administration (FDA) for clinical translation. Two primary obstacles may explain this discrepancy between scientific and clinical findings: (1) the ability of organisms to recognize and remove foreign substances via NP uptake by the reticuloendothelial system (RES), and (2) a complex circulatory environment with high levels of proteins and circulating immune cells in vivo, leading to interactions that further promote NP clearance [11].

Poly(ethylene glycol) (PEG) has been extensively employed as the gold standard means of modifying NP surfaces, allowing for a reduction in NP recognition by the immune system and thereby extending circulation time [12]. The PEGylated polymers used for coating NPs are able to create a hydration layer, which is known to markedly reduce rates of nonspecific interactions in the bloodstream and to suppress RES uptake, thus increasing NP uptake time in vivo from minutes (for uncoated particles) to hours (for PEG-coated particles) [13, 14]. However, PEGylation is an imperfect solution, with recent studies revealing that upon subsequent dosing PEG-coated NPs are rapidly cleared by the liver in a phenomenon referred to as “accelerated blood clearance (ABC)” [15]. Such rapid clearance is associated with both IgM antibodies specific for PEG, as well as with PEG-mediated complement activation that can drive hypersensitivity in some cases [16]. As a consequence, at present PEG is not well-suited to long term application. In addition, this “bottom-up” modification strategy, which requires pairing with the original group, is difficult to apply to large-scale production.

Effective drug delivery systems must allow for the shielding of cargo from rapid degradation, long-term in vivo retention, immune escape, controlled and targeted cargo release, and the ability to cross specific barriers in vivo [17]. In an effort to replicate mammalian physiology, there have been many recent efforts to produce biomimetic systems better suited to in vivo drug delivery [18]. Such cell biomimetic approaches include efforts to replicate the surface composition, shape, and movement of normal cellular physiology [19]. One of the most prominent approaches to NP functionalization relies upon the use of cell membrane coating [20]. Cell membrane coating technology is a simple top-down approach which utilizes cell membrane as a carrier facilitating the undetected targeted delivery of core NPs without specific regard to inner core nanomaterial properties [21]. As the membrane coatings are structurally and functionally similar to those of host cells, they can express specific markers useful for appropriate NP delivery. For example, CD47, an integral membrane protein expressed on red blood cells (RBCs) and platelets, functions as a “do not eat me” signal that prevents the macrophage-mediated clearance from circulation [22]. When NPs are encompassed in a natural cell membrane, additional external modifications are no longer required. To date, cell membrane coating approaches have sought to mimic the surfaces of bacteria, cancer cells, platelets, RBCs, stem cells, and leukocytes (Table 1). This coating strategy has been explored in fields including drug delivery, vascular injury repair, tumor imaging, optical therapy, detoxification, and immunotherapy.

**Table 1:** Summary of the differences among different cell membrane-coated NPs

Cell membrane type	Key features	Targeting ability	Stage of development	Limitations
Red blood cell membrane	Long systemic circulation (~ 120 d in human and ~ 50 d in mice) Immune evasion Surface expresses CD47 protein	RES-targeting	Clinical trial	Surface modification may induce hemolysis
Platelet membrane	Long systemic circulation (~ 7 to 10 d) Survey for damage Immune evasion Surface expresses CD47, CD55 and CD59 Self-aggregation Adhesion at tumor sites	Injury sites-targeting	Clinical trial	Small proportion of blood Undesirable activation
Leukocyte membrane	Amoeboid movement Close relationship with inflammation Immune evasion Endothelial adherence Solid and metastatic tumor interaction	Diseased sites-targeting	Lab study	The least component in blood Various subspecies with different morphology Limited to certain tumors
Cancer cell membrane	“Homologous adhesion” to tumor sites Drives tumor-specific immunity	Tumor-targeting	Lab study	Shorter circulation time
Stem cell membrane	Long circulation Tumor-specific properties	Tumor-targeting	Lab study	Low specificity
Fibroblast cell membrane	Homologous targeting ability	Cancer-associated fibroblasts-targeting	Lab study	Part targeting to normal fibroblast
Bacterial membrane	Stimulating innate immunity Promoting adaptive immunity	Homologous-targeting	Lab study	Need to remove peptidoglycan during extraction

The present review serves as an overview of recent advances in the development of cell membrane coating technology. Herein, we systematically describe the preparation process for membranes, and survey various cell membrane types that have been applied in the text of drug delivery system, phototherapy, immunomodulation, and detoxification. In addition, we summarize the model drugs used for studies of the cell membrane coating technologies. Furthermore, we compile the patent applications in this field over the past decade. Finally, we discuss the future directions of this technology.

## The Theoretical Basis for Cell Membrane Coating Technology

Cell membrane-coated NPs (CM-NPs) have recently been generated, fusing together the advantages of both host cells and artificial NPs [23]. The origin of the cell membrane coating technology can be traced back to 2011, when it was first reported by Zhang et al. [24], who took a top-down strategy that employed intact cell membranes to coat NPs. Compared with synthetic “stealth” particles, NPs coated with an RBC membrane exhibited a longer half-life in vivo in mice, with a retention time in circulation up to 72 h. This strategy relies on NPs being disguised by cell membranes, effectively allowing for these particles to interact with the surrounding environment through the use of translocation surface membrane components [25]. The resultant coated NPs have both the physical and chemical properties of the nanocarrier itself, as well as the biological properties of natural cells.

### Preparation

**Fig. 1:**
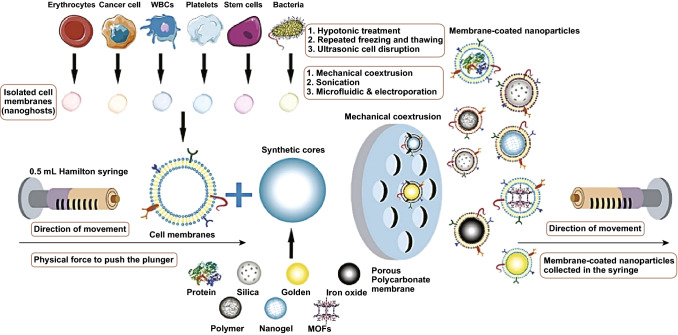
Membrane coating via physical co-extrusion approach. After obtaining appropriate cell membranes via hypotonic isolation, repeated freeze/thawing, or ultrasonic disruption, synthetic NP cores are co-extruded through a porous polycarbonate membrane. Adapted from Ref. [23] with permission

There are several extant approaches for fabricating CM-NPs. Conventional generation of CM-NPs can be separated into three key steps: membrane extraction, inner core nanocarrier production, and the fusion process (Fig. 1), each of which is the key to resultant NP functionalization.

#### Membrane Extraction

Cell membranes are composed of phospholipids embedded with specific surface proteins [26]. The membranes typically play central roles in a wide range of biological functions including transport, cell–cell recognition, and related processes [20]. The process of cell membrane extraction includes membrane lysis and membrane purification, both of which must be as gentle as possible [27]. The exact extraction process is determined by the cell type of interest.

For nucleus-free cells, such as mammalian mature RBCs and platelets, the process of membrane extraction is relatively simple. Initially, cells are isolated from whole blood using appropriate methodology, followed by either hypotonic lysis or repeated freeze/thaw cycles to mechanically disrupt membranes. Differential centrifugation then allows for the removal of soluble proteins, after which nano-vesicles are formed via extrusion [28, 29].

Eukaryotic cells, such as leukocytes, cancer cells, and stem cells, necessitate complex membrane extraction protocols. First, the target cells need to be isolated from tissues or blood, following by cell culture [30, 31]. A combination of hypotonic lysis, mechanical membrane disruption, and discontinuous sucrose gradient centrifugation is then used to remove the cell nuclei and cytoplasm to isolate cell membranes [32, 33]. Membranes are washed by isoionic buffers, followed by additional sonication and extrusion through a porous polycarbonate membrane [34].

#### Inner Core Nanocarriers

Inner core nanocarriers are important in generating CM-NPs, as they are the payloads ultimately delivered to targeted tissues [35]. In recent years, various type of materials (Table 2) for cell membrane encapsulation have been widely explored and applied, including poly(lactic-co-glycolic acid) (PLGA) [36], liposomes [37], SiO_2_ [38], mesoporous silica nanocapsules (MSNs) [39], gold [40], iron oxide [41], upconversion nanoparticles (UCNPs) [42], metal–organic frameworks (MOFs) [43], nanogels [44], and black phosphorus [45]. During preparation, the inner core nanocarrier should be selected according to the needs of the specific cargo delivery.

**Table 2:** Inner core nanocarriers for cell membrane coating approaches

Materials	Coating cell membrane	Features	Size (nm)	Zeta potential (mV)	References
PLGA	RBC membrane	FDA approved Good biodegradable Biocompatible Non-toxic High drug loading capacity	97.9	− 31.3	[52]
			121.0	− 48.3	[53]
	T Cell Membrane		88.3	− 49.2	[54]
	Macrophage membrane		84.5	− 41.3	[55]
	T Cell Membrane		42	/	[56]
	Cancer cell membrane		79.8	− 34.3	[57]
	Bacterial membrane		93.0	− 24.7	[58]
Liposomes	Macrophage membrane	Easy preparation Hydrophilic and hydrophobic cargo delivery	64.5	− 28.0	[32]
	RBC membrane		100	− 21.0	[59]
Silica/SiO_2_	RBC membrane	Easy preparation Good biodegradable	50	− 21	[38]
			120	20.9	[60]
	Cancer cell membrane		85.7	+35.4	[61]
			90.4	+32.7	[62]
MSN	RBC membrane	Large surface area Tunable pore sizes High pore volume	91.2	+5.1	[39]
UCNP	RBC membrane	Convert near-infrared (NIR) light into visible light Narrow emission peaks Low Toxicity Good photo-stability	30	− 5.89	[63]
			80	/	[42]
	Cancer cell membrane		80	/	[64]
Gold	RBC membrane	High photothermal conversion efficiency Excellent biocompatibility Tunable localized surface plasmon resonance (LSPR) peak	71.2	− 19.7	[65]
			70.1	− 42.2	[66]
	Platelet membrane		Length 50 nm Width 12 nm	+ 35	[67]
	Cancer cell membrane		82.3	− 19.7	[40]
	Bacterial membrane		30.3	− 38.6	[68]
Iron Oxide	Cancer cell membrane	Low toxicity Good biocompatibility High stability Capability as magnetic resonance imaging (MRI) contrast agents	285.6	− 4.4	[69]
	Myeloid-derived suppressor cell membrane		80	− 18	[70]
	RBC membrane		82.3	− 14.2	[71]
			172.3	− 14.4	[72]
			151	− 27.9	[41]
MOFs	Cancer cell membrane	High photosensitizers loading Facilitate intersystem crossing for PDT	126.9	+ 25.8	[49]
Nanogel	RBC membrane	High drug loading Have a macroporous structure	170	/	[44]
			130.2	− 23	[73]
			104	/	[74]
BP	RBC membrane	High photothermal conversion efficiency Excellent biodegradability	3	− 17	[45]
Bovine serum albumin	RBC membrane	Unique spatial structure Increase the solubility of insoluble drugs Protecting oxidizable drugs	67	− 23.1	[75]
Perfluorocarbon (PFCs)	RBC membrane	Highly hydrophobic and lowly reactive Have ability to dissolve large amounts of gases such as oxygen and carbon dioxide	380	− 50	[76]
			140	− 32	[77]
Nanocrystals	RBC membrane	High drug loading	80	− 18	[78]

Organic nanocarriers are made up of organic lipids and polymers. The US FDA has approved the use of PLGA for such purposes, and this compound is the most widely used to organic NP generation owing to its good biocompatibility and high drug loading capacity [46]. Liposomes are another kind of commonly-used nanocarrier, many of which have entered clinical trials and received FDA approval for specific clinical indications [47].

Inner core nanocarriers are also developed using inorganic materials, which are low in cost and easy to synthesize, and can be camouflaged with appropriate membrane vesicles. It is easy to better controlthe inorganic particles’ surface composition, shape, and size based upon specific optical, magnetic, or electrical properties [48]. Recently, several new inorganic materials have been used in cell membrane coating technology. Porphyrinic MOFs, including porous coordination network (PCN)-224, exhibit high loading capabilities for effective cytotoxic reactive oxygen generation in photosensitization applications. A study of cancer cell membrane-coated PCN NPs for tumor treatment has confirmed its effective functionality in the text of photodynamic therapy (PDT) [49]. Nanogel is also an ideal core material due to its macroporous structure and high loading capacity that required for multiple therapeutic strategies [50]. Black phosphorus (BP) has also been identified as an efficient photothermal therapy (PTT) agent for cancer therapy due to its excellent photothermal performance [51]. Negative potential and biodegradability enable BP to combine with cell membranes. Liang et al. [45] first reported BP-RBC membrane-mediated PTT together with antibody-mediated checkpoint blockade, thereby allowing for increased tumor infiltration and CD8^+^ T cell activity, constraining basal-like breast tumor growth in vivo.

#### Fusion Process

After obtaining the membrane and the inner core nanocarrier, these two materials must be fused so that the membrane can cover the surface of the core, yielding cell membrane biomimetic NPs. Currently, there are three fusion methods in use: membrane extrusion, ultrasonic fusion, or electroporation. Membrane extrusion and ultrasonic treatment are the two most frequently used methods in the literatures to date. For cell membrane extrusion, both membrane vehicles and inner core nanocarriers can be extruded for several times repeatedly through a nanoscale polycarbonate porous membrane using an Avanti mini extruder. During this extrusion process, mechanical forces lead to cell membrane coating of NPs [79]. This method is convenient and effective, but it is difficult to prepare on a large scale. When NP cores are co-incubated with membrane components and sonicated, this can similarly drive cell membrane-coated NP generation, although the resultant particles can vary significantly in terms of size and lack uniformity [80]. Recently, a novel microfluidic electroporation approach has been utilized for membrane-coated NP generation. The device first merges together components in a Y-shaped channel before mixing them thoroughly prior to electroporation. With appropriate optimization, this approach has been used to achieve efficient and reliable NP generation [81].

### Characterization

The evaluation of CM-NPs includes an assessment of their physicochemical and biological properties, in order to confirm that the cell membrane has been successfully coated on the NP surface. The success of cell membrane coating can be determined based upon NP size, surface charge, and protein composition.

**Fig. 2:**
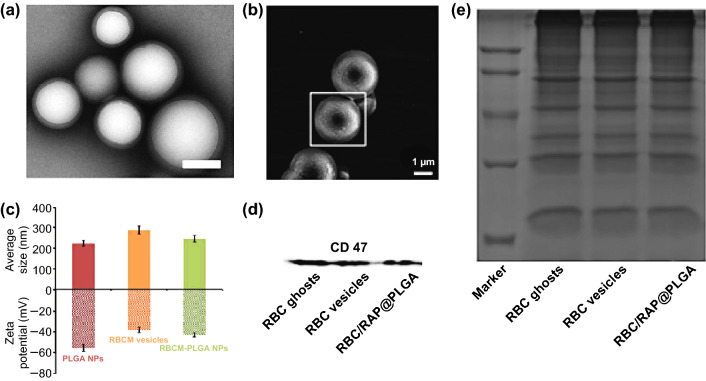
Cell membrane-coated NP characterization. **a** TEM image of RBCM-NPs showing the core–shell structure. Scale bar, 200 nm. Adapted from Ref. [36] with permission. **b** SEM images of the platelet membrane-derived vehicle-coated Si particles. Adapted from Ref. [82] with permission. **c** Average sizes and zeta potentials of PLGA NPs, RBC membrane vesicles, and RBCM-PLGA NPs. Adapted from Ref. [36] with permission. **d** CD47 levels measured by Western blotting demonstrating the retention of characteristic membrane proteins. **e** SDS-PAGE protein analysis of RBC-ghost, RBC-vesicle, and RBCM-NPs. Adapted from Ref. [83] with permission

Cell membrane coating alters both NP size and zeta potential, and transmission electron microscopy (TEM) images can be used to confirm the morphology of CM-NPs (Fig. 2a) [36]. TEM images of CM-NPs exhibit a roughly 20 nm increase in diameter compared with uncoated NPs. Scanning electron microscopy (SEM) can further be used as a means of examining membrane-coated NP morphology (Fig. 2b) [82]. Zeta potential offers information regarding the surface potential of particles prior to and following the coating process. For example, in one study, following RBC membrane coating the zeta potential of particles increased by ~ 10-mV (Fig. 2c) [36]. Particle size distributions can further be assessed via dynamic light scattering (DLS) measurements, with coated particles increasing in size relative to uncoated particles (Fig. 2c).

Physicochemical properties alone can confirm that coating was successful; the biomimetic function of the cell membrane depends on its biological characteristics. As such, verifying that this membrane is correctly oriented and biologically active is essential to ensure optimal NP coating efficacy. Western blotting is a common molecular biology technique useful for confirming protein expression in cells or other experimental systems, allowing researchers to confirm the presence of particular surface proteins on coated NPs. For example, the results shown in Fig. 2d confirmed the presence of CD47 (an RBC membrane marker) on the surface of the RBC membrane-coated NPs (RBCM-NPs) [83]. Relative protein levels in source cell membranes, membrane extracts, and coated NPs can thus be tested via SDS-PAGE, and in this instance levels were similar across samples (Fig. 2e), confirming that the membrane proteins on RBCM-NPs were consistent for all stages of fabrication.

In summary, the preparation and characterization of CM-NPs is a relatively well-developed process. As the demand for these particles and the scope of their utilization continue to develop, these methods are being further optimized. In addition, the types of materials used for the inner core particles continue to increase, utilizing different forms of liposomes, nanogels, nanoemulsions, and nanocrystals. With this continuing diversification of the inner core, the available means of characterization are becoming increasingly abundant (including strategies relying upon the ultraviolet and infrared spectra), in order to fully ensure that the preparation of CM-NPs is consistent with experimental expectations.

## RBC Membrane-Coated Nanoparticles

RBCs are the most prevalent form of blood cell in humans, and are essential for transporting oxygen from the lungs to distal sites via the hemoglobin protein contained within each cell. Normally, RBCs are 7–8 µm in diameter, and as thin as 1 µm in the center of each cell. RBCs also lack nuclei, and are able to undergo changes in shape while circulating through the body. In addition, RBCs can be easily isolated from donor blood, and they thus represent a potentially ideal source of cellular membranes well suited to in vivo circulation throughout the vasculature of patients [84]. RBCs express the self-recognition protein CD47 on their surface, and this protein is recognized by the reticular endothelial system (RES), allowing for long-term RBC circulation in vivo (~ 120 d in human and ~ 50 d in mice) [85]. When erythrocyte membranes are used to coat NPs, the resultant particles exhibit surface antigens consistent with a “self” identity, allowing these particles to circulate for longer without being recognized and eliminated by macrophages in vivo [86]. RBCs are completely biodegradable and nontoxic. In addition, as the membrane of RBCs is only semi-permeable, cargo release is gradual, thus ensuring that a sustained release can be achieved when utilizing RBCM-NPs [39].

### Drug Delivery

In the past 10 years, RBCs have been a major topic of research interest as a means of achieving effective drug delivery, owing to their excellent biocompatibility [87], limited immunogenicity [88], flexibility, and prolonged circulation [89]. In one recent report, mesoporous silica nanocapsule NPs coated with an RBC membrane were demonstrated to exhibit long-term circulation in the bloodstream, allowing for effective drug release and tumor imaging applications [39]. MSNs and RBC membranes in this study were co-loaded with the anticancer drug doxorubicin (DOX), with drug loading (DL%) and encapsulation efficiencies (EE%) of 39.8% and 97.6%, respectively. When NPs were coated using an RBC membrane, NP circulation time increased significantly, likely as a consequence of membrane-mediated immune evasion by these particles. The RBC membrane additionally ensured that DOX was not prematurely released from particles.

RBC membrane-encapsulated NPs are able to overcome certain drug limitations, such as poor water solubility or significant side effects. For example, Gambogic acid (GA) is a novel potential anticancer compound, but it is known to exhibit poor water solubility and to have a potentially high rate of adverse side effects, limiting its clinical utility. To overcome such limitations, Zhang and colleagues coated PLGA NPs with RBC membrane, and assessed whether such coating of GA-loaded NPs was compatible with drug retention and better GA antitumor efficacy [89]. In their study, they demonstrated that RBCm-GA/PLGA NPs not only achieved antitumor efficacy in vitro, but also inhibited subcutaneous tumor growth in vivo, caused tumor necrosis, and decreased tumor volume, whereas an equivalent dose of uncoated GA was only marginally able to control tumor growth, doing so far less effectively in vivo than in vitro. As such, the resultant biomimetic NPs were better able to exploit the antitumor properties of GA. In another example, Fu and colleagues developed a means of co-encapsulating paclitaxel (PTX) and DOX into magnetic O-carboxymethyl-chitosan NPs coated with RBC membrane [90]. They then evaluated potential PTX and DOX-associated side effects. They found that IgE levels in NP-treated groups tended to be normal, whereas Taxol^®^ or Taxol^®^/DOX markedly increased these levels. After treatment with free DOX, they also observed myofibrillar loss as well as cytoplasmic vacuolization, whereas animals treated with NP formulations of these drugs exhibited far lower rates of such outcomes. Importantly, these NPs also better allowed for the maintenance of normal myocardial morphology. Zhang’s group have found that supplementing RBC membranes with additional cholesterol can allow for better maintenance of a pH gradient in the resultant NPs, allowing for more effective DOX and vancomycin (Vanc) loading. When this group employed DOX-RBC particles as a means of treating breast cancer model mice, they found that the NPs effectively constrained tumor growth. Similarly, Vanc-RBC particles were effective means of reducing bacterial titers in a model of methicillin-resistant *Staphylococcus aureus* (MRSA) skin infection, preventing lesion formation completely over a 5-day period [91].

**Fig. 3:**
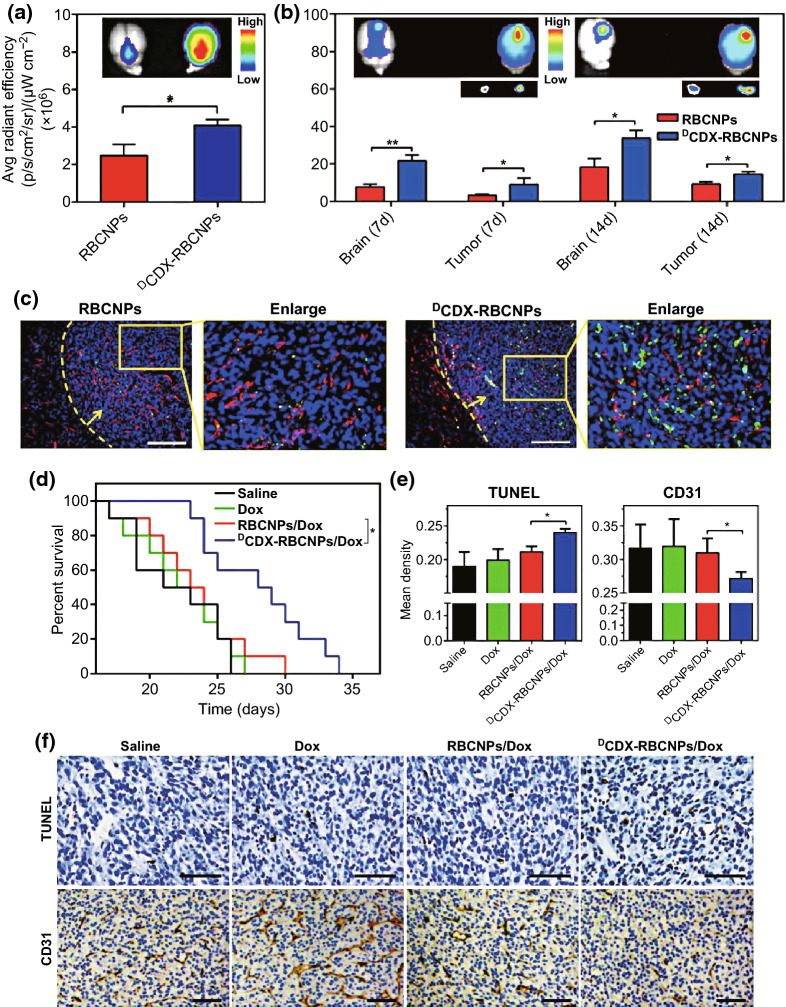
**a, b** Normal radiance values and images of the brains of normal nude mice and of brains bearing gliomas at days 7 or 14 of tumor progression. Mean ± SD, *n* = 3, *p < 0.05, **p < 0.005. **c** NP localization on day 14 post-tumor implantation, with DAPI (blue) representing nuclear staining, CD31 (red) indicating vasculature, and green indicating NPs. Glioma margin is demarcated by a yellow dotted line, and the tumor is indicated by a yellow arrow; Scale bar = 200 µm. **d** Survival of intracranial U87 glioma-bearing mice calculated via the Kaplan–Meier method. On days 7, 9, 11, 13, and 15 following tumor implantations, animals (*n* = 10) were injected using either saline as a vehicle control, or with free DOX, DOX-loaded RBC NPs (RBCNPs/DOX), and DOX-loaded ^D^CDX-RBC NPs (^D^CDX-RBCNPs/DOX). **e** Integral optical density (IOD) values for tumors from differentially treated mice following either TUNEL staining or CD31/PAS dual staining. Mean density = IOD (SUM)/Area. Mean ± SD, *n* = 3, *p < 0.05. **f** TUNEL staining and CD31/PAS dual staining analysis of tumors. Scale bar = 100 µm. Adapted from Ref. [92] with permission

RBCM-NPs offer many advantages, but in order to achieve effective targeting, some functionalization measures need to be taken. Recently, an RBC membrane-coated solid lipid NP was developed and modified to contain the T7 and NGR peptides [88]. When loaded with vinca alkaloid vincristine (VCR), the resultant particles achieved effective anti-glioma efficacy both in vitro and in vivo owing to their effective dual targeting efficacy. Similarly, Zhang et al. [87] have modified RBCM-NPs by employing a lipid insertion approach to add recombinant anti-EGFR-iRGD to the particle surface, allowing them to achieve successful and accurate tumor-targeting in a high EGFR-expressing colorectal cancer model, whereas NPs without peptide modification were less efficacious. The particles could also be loaded with GA, leading to better anti-tumor efficacy than that of free GA. Chai and colleagues similarly utilized a targeting moiety derived from a neurotoxin in order to develop modified RBC NPs [92]. They conjugated biotin to the CDX peptide (^D^CDX), and then used avidin to bind this peptide to the surface of the RBC NPs, with the resulting particles achieving significantly higher brain distributions (Fig. 3a). Specifically, the ^D^CDX-RBC NPs were present at markedly higher levels in the cortical, hippocampal, ventricular, and corpus striatal regions. When used in mice bearing an intracranial U87 glioma model tumor, the investigators were able to assess the distribution of the resultant NPs over the course of tumor progression (Fig. 3b). At all assessed time points, the investigators found that the ^D^CDX modification resulted in higher levels of fluorescence in the brain and tumor. The investigators further utilized frozen tumor sections to assess NP distribution in vivo in a more accurate manner (Fig. 3c). The ^D^CDX-RBC NPs were ultimately distributed at higher levels in glioma tissues, and were primarily restricted to the tumor rather than to the surrounding vasculature. Importantly, the modified NPs were able to prolong murine median survival by 4.3-fold relative to mice treated with RBC NPs lacking the ^D^CDX modification (Fig. 3d). When the particles were loaded with DOX, ^D^CDX-RBC NPs induced significantly higher levels of apoptosis and reduced angiogenesis more effectively than did unmodified RBC NPs loaded with DOX (Fig. 3e, f). The results of this study thus confirm that ^D^CDX modification of RBC NPs may be an effective glioma treatment strategy owing to the ability of the peptide to mediate drug delivery over the blood brain barrier.

RBC membranes have also been used to achieve better glucose-responsive insulin delivery to patients. For example, Gu et al. [93] determined that RBC membranes can effectively bind to insulin that had been modified using a derivative of glucose (termed Glc-Insulin). In a murine model of inducible type 1 diabetes, they found that RBC membrane-coupled Glc-insulin had a longer half-life following intravenous injection in vivo, improving the maintenance of normal blood glucose levels. This may be associated with the presence of glucose transporter (GLUT) molecules on the RBC surface, thus allowing for reversible interactions between Glc-insulin and RBC membranes such that in the presence of high glucose levels, insulin molecules are released, as free glucose undergoes a competitive interaction with GLUTs.

RBC membranes are biogenic, and they have potential to replace PEG in some contexts and to overcome drug limitations. At present, an extensive body of research has focused on the development of RBCM-NPs, with increasingly advanced particles being developed regularly.

### Phototherapy

**Fig. 4:**
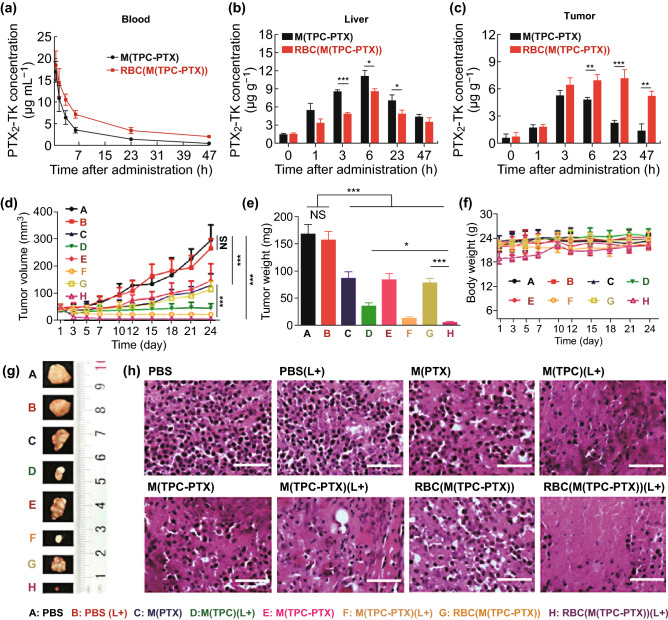
**a** Mice (*n* = 4) were i.v. administered drug-loaded NPs with or without an RBC membrane coating, using a 15 mg kg^−1^ PTX equivalent dose. PTX_2_-TK levels in **b** the liver and **c** tumor were assessed at specific time points following NP administration. **d** Tumor volume changes in mice treated with a range of formulations (PBS, PBS (L+), M(PTX), M(TPC) (L+), M(TPC-PTX), M(TPC-PTX) (L+), RBC(M(TPC-PTX)) and RBC(M(TPC-PTX)) (L+)). L+ indicated laser. **e** Tumor weights of mice treated as in **d**. **f** Body weights of mice over time. **g** Ex vivo tumor images, as indicated. **h** Tumor sections were H&E-stained following the indicated treatments. Scale bar = 200 µm. Data **d-g** are mean ± SEM (*n* = 6). *p < 0.05, **p <0.01, and ***p <0.001. Adapted from Ref. [95] with permission

Phototherapy is a noninvasive approach to treating cancer, and encompasses techniques including PDT and PTT [77]. During cancer therapy, PDT and PTT have shown promising utility while inducing minimal side effects and maintaining high selectivity [94]. PDT has been exploredto treat a wide array of disease types [63]. Pei et al. [95] developed NPs coated with RBC membrane that mediated drug release in response to light, allowing for synergistic PDT-mediated chemotherapy. An inner core NPs was composed of a combination of a reactive oxygen species (ROS)-sensitive PTX dimer (PTX_2_-TK) as well as a photosensitization agent (5,10,15,20-tetraphenylchlorin (TPC)). When studied in vitro, the resultant NPs were readily internalized into the endosomes of cells, and the circulation of these RBC membrane-coated NPs was extended by the coating process, with reduced liver uptake consistent with these particles being recognized as “self” to avoid RES-mediated liver uptake (Fig. 4a, b). Importantly, RBC membrane coating of NPs led to higher concentrations of PTX_2_-TK in the tumor tissue with maximal doses achieved 23 h following administration (Fig. 4c). Exposure to the proper light source was able to trigger ROS generation for PDT, as well as cleavage of the PTX_2_-TK molecule to release chemotherapy drugs in a controlled manner. In this study, researchers used a murine human cervical carcinoma model system to assess the in vivo efficacy of the particles in nude mice. Mice were i.v. administered a range of formulations containing PTX (30 mg kg^−1^) and TPC (10 mg kg^−1^), and after 6 h appropriate animals underwent a 15-min irradiation step using a 638 nm laser lamp (200 mW cm^−2^). This led to some inhibition of tumor progression, indicating that PDT and chemotherapy can be effectively combined in vivo (Fig. 4d). Importantly, drug-loaded NPs coated with RBC membrane achieved the most profound anti-tumor efficacy in animals, owing to their extended circulation and preferential accumulation within tumors. Excised tumor volumes (Fig. 4e, g) were consistent with findings in vivo (Fig. 4f). Animal body weights were unchanged by treatment, suggesting that none of the NPs were inherently toxic (Fig. 4f). Additionally, stained tumor sections revealed that nuclear ablation was best achieved in animals treated using drug-loaded RBC membrane-coated particles (Fig. 4h).

In a related approach, Xuan et al. [96] produced RBC membrane-coated mesoporous silica NPs to deliver photosensitization agents, and they combined the particles with magnetic targeting in order to enhance PDT efficacy. In this approach, mechanically separated RBC membrane vesicles were used to coat mesoporous silica NPs that had been loaded with the PDT agent hypocrellin B (HB). They found that RBCM-NPs were able to circulate significantly longer in vivo, and represented a viable means of HB delivery. By combining the particles with magnetic field-mediated targeting and appropriate light irradiation, the authors demonstrated the ability of the RBCM-NPs to mediate effective HB accumulation in tumors, thereby enhancing anti-tumor efficacy and constraining growth of the 4T1 tumor model in mice.

**Fig. 5:**
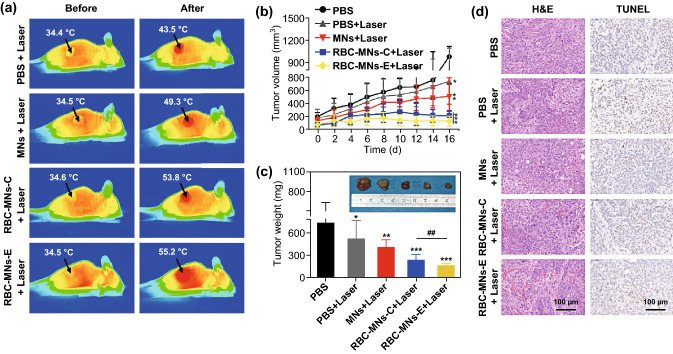
**a** Infrared imaged from representative mice before and after treatments, with black arrows indicating tumor sites. **b** Tumor volumes over time in treated animals. **c** Tumor weights in differentially treated animals, with inset images displaying representative ex vivo tumor images at the end of treatment. **d** Representative H&E- and TUNEL-stained images from differentially treated mice. Data are mean ± SEM (*n* = 6). *p < 0.05, **p < 0.01, and ***p < 0.001 relative to control, respectively, and## represents *p *< 0.01 relative to conventional extrusion method. Adapted from Ref. [97] with permission

Liu et al. [97] utilized magnetic RBCM-NPs generated via a microfluidic electroporation approach to achieve good PTT treatment efficacy. They employed a microfluidic chip to achieve successful electroporation-mediated fusion of RBC membrane vesicles and NPs, allowing for the production of particles which could then be administered to BALB/c nude mice implanted with the human breast MCF-7 tumor cell line. They found that the RBCM-NPs were able to accumulate in tumors better owing to an enhanced permeability and retention (EPR) effect. When animals were treated via a 1-h laser irradiation following administration of differently prepared NPs, the authors found that the tumor temperature in mice treated using electroporation-generated RBCM-NPs rapidly rose over a 5-min period from 34.5 to 55.2 °C following laser treatment (Fig. 5a). Tumor growth was almost completely inhibited in these animals (Fig. 5b), and they achieved the best tumor inhibition of all treated groups (Fig. 5c). H&E and TUNEL-staining (Fig. 5d) further confirmed that PTT following treatment with the NPs led to a near complete destruction of tumor tissues, with extensive cell necrosis and apoptosis.

One recent PTT strategy has focused on using RBC membranes to coat nanorods [98]. When animals bearing tumors were treated with the resultant biomimetic nanorods and laser irradiated, animals in a group that received cyclopamine achieved the highest average temperature (57.2 °C) at the tumor site, as compared to 48.5 °C in control animals. Tumor size reductions were also most significant in the treated mice (Tumor growth inhibition rates based on tumor size (TGIRv): 80.60 ± 0.21%; Tumor growth inhibition rates based on tumor weight (TGIRw): 81.1 ± 0.151%) bearing Capan-2 xenografts. In a separate study, Liu et al. [99] coated gold nanocages with RBC membranes. They modified RBC membranes with antibodies specific for EpCam, thereby allowing for targeting of the anti-cancer drug paclitaxel to 4T1 tumor cells following gold nanocage encapsulation. Following appropriate laser irradiation (808 nm; 2.5 W cm^−2^), the tumor temperature rose to 49 °C in animals treated with these particles within 5 min, whereas PBS-treated control animals exhibited only a 3 °C increase. This localized hyperthermia was able to release PTX owing to heat-mediated RBC membrane disruption, and directly damage surrounding tumor cells.

Both PDT and PTT together represent a viable strategy for enhancing anticancer therapeutic activity. Ren et al. [100] generated an RBC membrane-coated oxygen-enriched biomimetic particles for PTT. They prepared HAS NPs containing a near infrared (NIR) dye and perfluorotributylamine (PFTBA), and the NPs were enclosed in an RBC membrane. Following irradiation with a NIR laser (808 nm, 1 W cm^−2^, 3 min), the particles were able to achieve a 62% tumor inhibition rate for PDT alone, and a 93% rate when PDT and PTT were combined. In a similar strategy, RBC-coated particles have been generated that target tumors and contain a bovine serum albumin (BSA) core with 1,2 diaminocyclohexane-platinum (II) (DACHPt) and indocyanine green (ICG), which are surrounded by an RBC membrane modified with appropriate targeting peptides [75]. The resultant particles were able to specifically target and ablate B16F10 tumors and prevent lung metastases from developing in vivo via the combination approach of PDT and PTT.

It is clear that phototherapy research in the context of RBCM-NPs is largely tumor-focused, and the prolonged circulatory characteristics of RBCs have been of great value in this context. RBC membranes are the first choice for bionic medical applications of new materials (e.g., BP) owing to their relatively easy extraction procedures. RBC membrane coating is thus the first step towards the development of biomimetic membrane-coated NPs. In addition, targeted modifications can allow RBCM-NPs to be further functionalized so as to achieve better therapeutic efficacy, and at present the combination of various therapeutic strategies represents a major area of research in the field of RBCM-NPs.

## Platelet Membrane-Coated Nanoparticles

Platelets are cells that arise from megakaryocyte progenitor cells [101]. They are vital blood components, participating in a wide range of processes including immunity, wound healing, and the metastasis of tumors [102]. Platelets are produced in large quantities in humans with a size of approximately 1–3 mm in diameter, surviving for 7–10 days on average in circulation [103, 104]. Platelet membranes offer potential advantages for NP coating, as they can mediate immune evasion through both CD47-mediated macrophage evasion and CD55/59-mediated avoidance of complement activation [102], with the latter two receptors being regulators of the complement cascade [105]. The platelet CD44 and P-selectin receptors can also allow them to bind to circulating tumor cells [106]. They can further indirectly interact with tumors and other cells via release factors that can promote aggregation including MMP-2 and thromboxane-A2 (TXA2), and in tumors, platelets have been shown to mediate tumor-induced platelet aggregation in a manner dependent upon the GPIb and GPIIb/IIIa receptors [107].

### Drug Delivery

Platelet membranes offer an opportunity to achieve NP-mediated drug delivery in a fashion which can specifically target tumor cells while evading immune detection [108]. As an example of this approach, Hu et al. [106] produced platelet membrane-coated nanovesicles (PMNVs) capable of delivering DOX and tumor necrosis factor (TNF)-related apoptosis inducing ligand (TRAIL) to target cells. Specifically, these PMNVs delivered TRAIL to the membranes of MDA-MB-231 cells, thereby inducing their extrinsic apoptotic death. PMNVs further contained an acid-sensitive matrix that was degraded upon particle endocytosis, leading DOX to be released into cells to promote further apoptotic death via the intrinsic pathway. As metastatic tumor cells in particular depend upon platelet-mediated aggregation for their ability to spread through their body, such PMNVs offer an opportunity to specifically target cancer cells with metastatic potential. In a separate study, researchers developed platelet membrane-coated NPs via surrounding DOX and melanin NPs (MNPs) with platelet vesicles modified using the RGD peptide (RGD-NPVs) [109]. The resultant particles were able to both evade immune-mediated elimination and target tumor vasculature-associated αvβ3 integrin expression as well as resistant MDA-MB-231 tumor cells, inhibiting drug-resistant breast cancer (MDA-MB-231/ADR) growth via this dual-targeting approach. Liu et al. [110] similarly used cholesterol-enriched platelet membranes as a means of encapsulating DOX and Vanc with a high rate of encapsulation efficiency, thereby allowing for effective drug delivery approaches. These particles had a natural affinity for 4T1 breast cancer cells and methicillin-resistant *Staphylococcus aureus*, offering an opportunity to enhance their ability to target disease in vivo, thus making the drug payloads more potent than untargeted free drug.

**Fig. 6:**
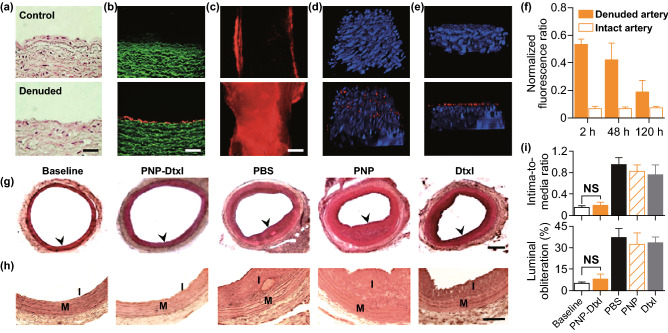
**a** H&E-stained human carotid arterial cross-sections from intact (upper) or injured (lower) samples. Scale bar = 200 μm. **b** Fluorescence imaging of cross sections (scale bar = 200 μm) and **c** luminal side (Scale bar = 500 μm) of intact (upper) or injured (lower) arterial tissue (in green) following incubation with PNPs (in red). **d**–**e** 3D reconstructions based on multiple section images from either undamaged (upper) or balloon-denuded (lower) rat arterial walls following i.v. PNP delivery, with nuclei stained blue and PNPs in red; 152.5 × 116 × 41 μm^3^. **f** PNP retention in denuded and undamaged arterial sites for 120 h following PNP administration (*n* = 6). **g** Representative arterial cross-sections from coronary restenosis model rats exposed to different treatments. Scale bar = 200 μm. **h** Magnified cross-sections highlighting differences in vascular remodeling among groups; I, intima; M, media. Scale bar = 100 μm. **i** Quantification of the intima-to-media area ratio and luminal obliteration in differently treated animals (*n* = 6). Data are mean ± S.D. NS, no significant difference. Adapted from Ref. [111] with permission

Platelets are able to bind to the subepithelial collagen exposed upon epithelial damage, and this binding has been leveraged to treat coronary restenosis–a condition that restricts arterial blood flow due to intima overgrowth following injury [25]. Recent efforts to produce platelet-mimetic NPs (PNPs) have exhibited increased DOX and Vanc efficacy when these PNPs were used for drug delivery in rodent models of coronary restenosis and bacterial infection, respectively [111]. Further testing of the particles was conducted using a human carotid artery segment in which the subendothelial layer was exposed via wounding (Fig. 6a). When arterial samples were exposed to fluorescent PNPs, cross section imaging revealed these particles to more readily adhere to the wounded tissue samples than to intact ones (Fig. 6b, c), with additional binding to the edges of intact samples at sites where subendothelial tissue had been exposed during tissue isolation (Fig. 6c). This in vitro result was consistent with results from an in vivo rat angioplasty-induced arterial injury model, wherein 2 h following PNP administration there was evidence of selective binding specifically to denude arterial tissue regions (Fig. 6c). PNPs localized to the luminal smooth muscle layer (Fig. 6e) for a minimum of 5 days on average following treatment (Fig. 6f). When rats were treated with PNP-Dtxl particles, neointimal growth was markedly inhibited based on arterial cross-sections collected 14 days following injury (Fig. 6g, h), with PNP-Dtxl yielding a markedly lower intima-to-media ratio (I/M) and luminal obliteration relative to free drug (Fig. 6i). As such, these findings clearly demonstrate that PNPs can be used to effectively mediate drug delivery in the context of vascular disease.

A separate group sought to use platelet membrane-coated polymeric nanoclusters to target injured arterial wall at the site of restenosis [112]. In this study, researchers loaded these nanoclusters using an epigenetic inhibitor (JQ1) known to protect the endothelium, or using rapamycin which is known to be toxic to the endothelium, and they then compared their ability to impair restenosis without disrupting endothelial healing. The platelet-coated nanoclusters were home specifically injured and not uninjured arterial sites, and at 2 weeks post-angioplasty, both of the drug-loaded biomimetic nanoclusters significantly reduced neointimal hyperplasia by more than 60% relative to controls.

Platelets are intrinsically capable of binding plaques and homing to regions of atherosclerosis, potentially making them viable for the treatment of such disease. Song et al. [113] have recently utilized PNPs with a PLGA core containing rapamycin to target drug delivery to atherosclerotic plaques. They found that the PNPs exhibited an almost fivefold increase in radiant efficiency relative to control uncoated particles, confirming the ability of the platelet membrane to mediate atherosclerotic plaque homing in vivo. They further observed a significant reduction in atherosclerotic progression in apolipoprotein E-deficient (ApoE^−/−^) model mice administered the rapamycin PNPs, with improved plaque stabilization. Together, the findings thus clearly indicate that platelet membrane-coated NPs represent a potential strategy for targeting and treating atherosclerosis.

Relative to RBCs, platelets are better suited to target injured tissues and tumor sites. Platelet membrane-coated NPs represent an ideal approach to drug delivery owing to their durable in vivo circulation and effective targeting to specific sites. This approach offers an new opportunity for the treatment of vascular diseases, including both restenosis and atherosclerosis.

### Phototherapy

**Fig. 7:**
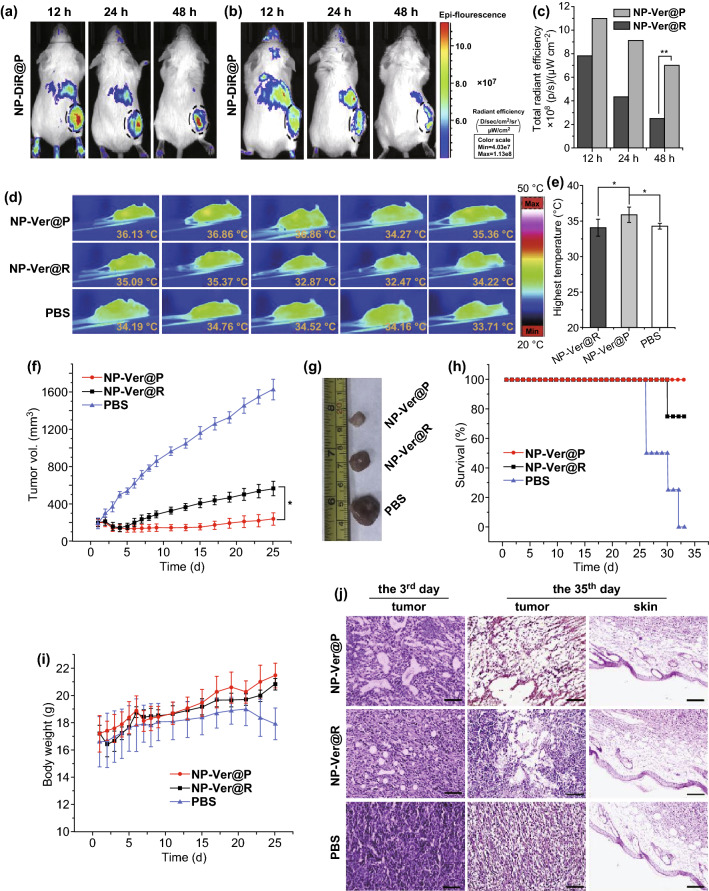
Mice received an i.v. injection of NPs, and after 12, 24, and 48 h, tumors and organs of random mice were isolated and used to assess fluorescence and verteporfin levels therein following homogenization. **a**–**c** In vivo fluorescence images of mice implanted with 4T1 tumors 12, 24, and 48 h following injection of **a** PNPs and **b** RBC-coated NPs loaded with the red membrane dye DiR, with black circled dots identifying tumors. **c** Total radiant efficiency in tumors was determined based on the images from **a** and **b**. **d** Mice (*n* = 5/group) were irradiated with 680–730 nm light (0.05 W cm^−2^) for 10 min, 24 h after administration of PBS or of PNPs or RBC-coated NPs loaded with verteporfin. **e** Quantification of average tumor center temperatures from **d**. Data are mean ± S.D. **f** Average tumor volume in mice following light irradiation. **g** Tumors were excised from treated mice 35 days following NP administration. **h** Mouse survival and **i** average body weight of differently treated mice following light irradiation. Mice treated using RBC-coated NPs loaded with verteporfin are included for reference, as are PBS-injected controls. Data are mean ± standard deviation. **j** Tumor and skin sections stained with H&E on days 3 and 35 after NP administration. Scale bar = 50 µm. *p < 0.05 and **p < 0.01. Adapted from Ref. [114] with permission

In addition to their utility for traditional drug delivery, PNPs may also represent ideal mediators of more effective PDT. Xu et al. [114] developed a PNPs containing verteporfin which serves as a sensitization agent, demonstrating that loading PLGA PNPs with verteporfin altered its absorption peak from 682 to 712 nm, thereby better facilitating penetration of deeper tissue. When the particles were compared in vivo in a murine 4T1 tumor model, they found that there was a much stronger fluorescent signal in mice treated with the PNPs relative to mice treated with comparably-prepared NPs instead coated with RBC membrane (Fig. 7a–c). Following a 10-min irradiation period (680–730 nm) 1 day following NP administration, infrared thermographs (Fig. 7d) revealed an elevated 35.9 °C local temperature in PNP-treated mice (Fig. 7e), likely as a result of the better tumor accumulation of these particles. Using Ver-loaded particles, they then assessed the relative utility of PNPs and RBC-coated NPs for tumor treatment, irradiating mice as above (680–730 nm; 0.05 W cm^−2^) daily for 3 days and then monitoring mice for up to 25 days. The average tumor volume of mice treated with these Ver-PNPs significantly decreased during the 4 days following treatment, with sustained suppression of tumor growth through the remainder of the study period (Fig. 7f). Consistent with this, following animal sacrifice at the study end, tumors from PNP-treated mice were smaller than those from other treatment groups (Fig. 7g). Importantly, 100% of mice in the Ver-PNP group survived during the 35-day study period (Fig. 7h). When tissue sections were collected from tumors of mice on the third day of treatment, H&E staining revealed there to be more significant lesion formation in mice treated with Ver-PNPs relative to other mice, and consistent findings were evident on day 35 when mice were sacrificed (Fig. 7i). Both Ver-PNP and control mice showed no evidence of skin tissue damage near the tumor site, suggesting that no photo-induced skin damage was induced (Fig. 7j).

As platelets could target lesions in vivo, this offers a novel strategy for PTT treatment. Liu et al. [67] have developed a platelet mediated tumor therapy strategy wherein the used PLTs to serve as a means of targeting photothermal compounds to tumor sites, thereby enhancing PTT efficacy. To this end, they loaded PLTs with gold nanorods via electroporation, yielding molecules which offered the advantageous photothermal properties of these gold nanorods together with prolonged in vivo circulation. When the resultant PLTs were administered to mice, the authors found local irradiation to be able to inhibit local head and neck squamous cell carcinoma (HNSCC) growth. Mice treated with the PLTs also exhibited the most significant increase in temperature following irradiation, likely due to the ability of these particles to circulate for extended periods and to effectively target tumors in vivo. Importantly, temperature rose following each treatment, suggesting that following PTT ablation, tumors attracted further nanorod-containing PLTs, creating a positive feedback loop useful for PTT-mediated tumor destruction.

**Fig. 8:**
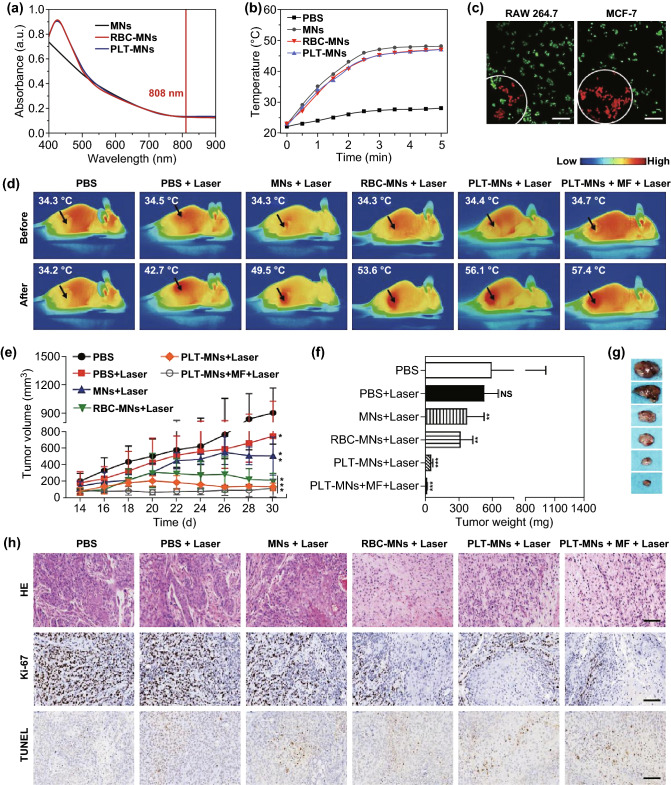
**a** NP UV–Vis absorption spectra. **b** Temperature change for control or NP preparations following 808 nm laser irradiation. **c** CLSM results from RAW 264.7 and MCF-7 cells following PLT-MN treatment and laser irradiation. FDA (green) and PI (red) were used to detect live and dead cells, respectively. Scale bar = 100 µm. **d** Representative in vivo IR thermal images from mice implanted using MCF-7 tumors following the indicated treatments. **e** Tumor volumes and **f** average weight following the indicated treatments. **g** Representative ex vivo tumor images and **h** HE-, Ki-67-, and TUNEL-staining of tumor tissue from differently treated animals. Scale bars = 200, 50, and 50 µm, respectively. Data are mean ± SEM (*n* = 6). NS: no statistical difference, *p < 0.05, **p < 0.01, ***p < 0.001, relative to PBS control. (Color figure online) Adapted from Ref. [115] with permission

Liu et al. [115] also demonstrated the ability of PNPs to improve cancer diagnostics. To achieve this, they coated Fe_3_O_4_ magnetic NPs (MNs) with murine platelet membrane vesicles, yielding PLT-MNs useful both for MRI and PTT. These PLT-MNs still absorbed UV light at ~ 808 nm, consistent with their potential for PTT utility (Fig. 8a). Following irradiation, it was clear that both MNs and PLT-MNs achieved comparable efficacy, confirming that PLT coating does not interfere with PTT efficacy in vitro (Fig. 8b). The authors also demonstrated that PLT-MNs were better able to achieved MCF-7 cancer cell killing at the site of laser irradiation than were MNs, owing to the selective binding of PLT-MNs to tumor cells (Fig. 8c). After mice bearing the MCF-7 tumors were treated using PLT-MNs and laser irradiation, there was an increase in the temperature of the tumor from 34.4 to 56.1 °C within 5 min (Fig. 8d), and there was also a 1.3 °C increase following magnetic field application, consistent with PLT-MN magnetic targeting. Tumor volumes and weights were also monitored in these mice, revealing near complete tumor ablation in mice treated using a combination of PLT-MNs, magnetic fields, and laser irradiation (Fig. 8e-g). Consistent with this, histological findings clearly indicated impaired tumor growth following this combination of treatments (Fig. 8h).

While PNPs have already been shown to be effective for PTT and PDT, some of the materials used for the inner core of such molecules may be limited in their utility as a consequence of hypoxic tumor environments and susceptibility to oxidative damage, necessitating a combination of PDT and PTT. In an effort to enhance PDT efficacy and to overcome tumor hypoxia, Zuo et al. [116] developed a novel PNP drug delivery system wherein W_18_O_49_ NPs and metformin (Met) were loaded into PNPs to allow for simultaneous PDT and PTT mediated by these two respective compounds. When Raji cells were combined with these PNPs and irradiated via 808 nm laser for 10 min, viability markedly decreased, and co-loading of Met greatly improved PDT efficacy. Rates of apoptosis were much higher when cells were treated with PNPs containing W_18_O_49_ with Met (88.30% apoptotic) or without Met (52.97% apoptotic) as compared to free W_18_O_49_ alone. In addition, generation of ROS and heat were markedly enhanced by these PNPs in vitro, and in vivo these dual-loaded PNPs markedly enhanced the therapeutic efficacy of these compounds, impairing Raji tumor growth and increasing rates of apoptosis.

Phototherapy is a major area of active cancer therapy research. The adhesive properties of platelets offer an opportunity to overcome the limitations of the uneven distribution of photosensitizers and photothermal converters in the context phototherapy. PTT relies upon thermal damage inducing cancer cell death, and this feedback after injury can facilitate passive platelet targeting, leading to their additional recruitment and enhancement of the photothermal effect. Thus, combining platelet membrane coating and PDT/PTT offers an opportunity to enhance the utilization of PTT in the treatment of cancer.

## Leukocyte Membrane-Coated Nanoparticles

Leukocytes, or white blood cells (WBCs), are immunological cells essential for defending hosts against pathogen invasion and disease [117]. These cells are significantly larger than RBCs, moving in an amoeboid fashion that allows for their rapid and effective extravasation from the blood into surrounding tissues, leading the cells to be abundant both in circulation and in extravascular sites [118]. There are five primary classes of leukocytes: lymphocytes, monocytes, neutrophils, eosinophils, and basophils. Relative to RBCs and platelets, WBCs are more complex and are nucleated, making the isolation of their membranes a more significant challenge. WBCs are closely tied to inflammatory processes and pathogen control, with cells such as monocytic macrophages serving to consume debris and microbial pathogens directly, whereas other cells rely on the release of cytotoxic and lytic compounds to destroy these pathogens [119]. Distinct leukocyte types are involved in the pathogenesis or prevention of specific diseases, with, for example, chronic inflammation being primarily associated with monocytes (e.g., macrophage cells) and lymphocytes (e.g., T cells, B cells, and NK cells), and acute inflammation being primarily associated with granulocyte activity. WBCs also exhibit unique adhesive and homing properties that allow them to interact with tumor cells both in the tumor site and in circulation [120].

### Drug Delivery

In order to efficiently deliver drugs in an efficacious manner, the drugs must evade phagocytic uptake by monocytic cells such as macrophages, while targeting the site of interest and bypassing any endothelial barriers to reach this target tissue. As such, many efforts to utilize WBC membrane-coated NPs have been developed to overcome these challenges. For example, WBC membrane-coated NPs (WBC-NPs) coated using J774 cell membranes were taken up 75% less by live J774 cells, while they were still able to specifically bind to inflamed epithelial sites and to facilitate transport of DOX across the endothelium without resulting in its lysosomal uptake [121]. Separately, Cao et al. [32] found that using RAW264.7 macrophage membranes to generate WBC-NPs containing the anti-cancer agent emtansine in pH-sensitive liposomes was able to enhance drug delivery to tumor metastatic sites. The macrophage-coated NPs were taken up more efficiently by 4T1 breast cancer cells than that uncoated particles, and in vivo the particles were able to inhibit 4T1 lung metastases by 87.1%—an improvement 1.88-fold higher than that of uncoated emtansine liposomes. Similarly, another study of DOX-containing NPs coated with U937 cell membranes exhibited significantly increased DOX loading into cancerous HeLa cells relative to uptake in healthy HEK293 cells [122].

WBC-NPs are a viable tool for mediating the long-term sustained release of drugs in vivo, as evidenced by a study in which PLGA NPs were coated in a membrane derived from monocytic U937 cells [123]. The resultant WBC-NPs were highly stable in serum for 120 h, and had a DOX loading efficiency of 21% with sustained drug release over a 72-h period. In a test of drug uptake by cells, the authors found that DOX uptake and associated cytotoxicity was greater when Dox-loaded WBC-NPs were used than when uncoated DOX-loaded PLGA NPs were used to treat MCF-7 breast cancer cells, likely owing to the improved tumor targeting, binding, and uptake of these coated NPs.

Recent efforts have employed the use of T cell-derived membranes for biomimetic drug delivery, with one study utilizing cytotoxic CD8^+^ T cell membranes to coat PLGA NPs [124]. This approach was combined with localized low-dose irradiation (LDI) as a means of mediating NP chemoattractant targeting. The resultant particles had a 23.99% reduction in macrophage uptake, and when used to deliver paclitaxel in vivo in a model of human gastric cancer, these particles were associated with a 56.58% inhibition of tumor growth. When used in combination with local tumor LDI, these particles achieved an even higher 88.5% inhibition of tumor growth.

NK cells are a form of lymphocyte able to directly interact with cancer cells through specific inhibitory and activating cell surface receptors, allowing for superior tumor targeting. Arunkumar et al. [125] sought to leverage this property, coating DOX-loaded liposomes with NK cell membranes to achieve effective tumor targeting. The resultant “NKsomes”, exhibited a higher affinity for tumor cells relative to normal healthy cells in in vitro assays, and this result was confirmed in vivo wherein the particles were able to persist in circulation for 18 h. In the MCF-7 tumor model, the NK some particles showed promise as a means of effectively and specifically delivering DOX to cancer cells.

**Fig. 9:**
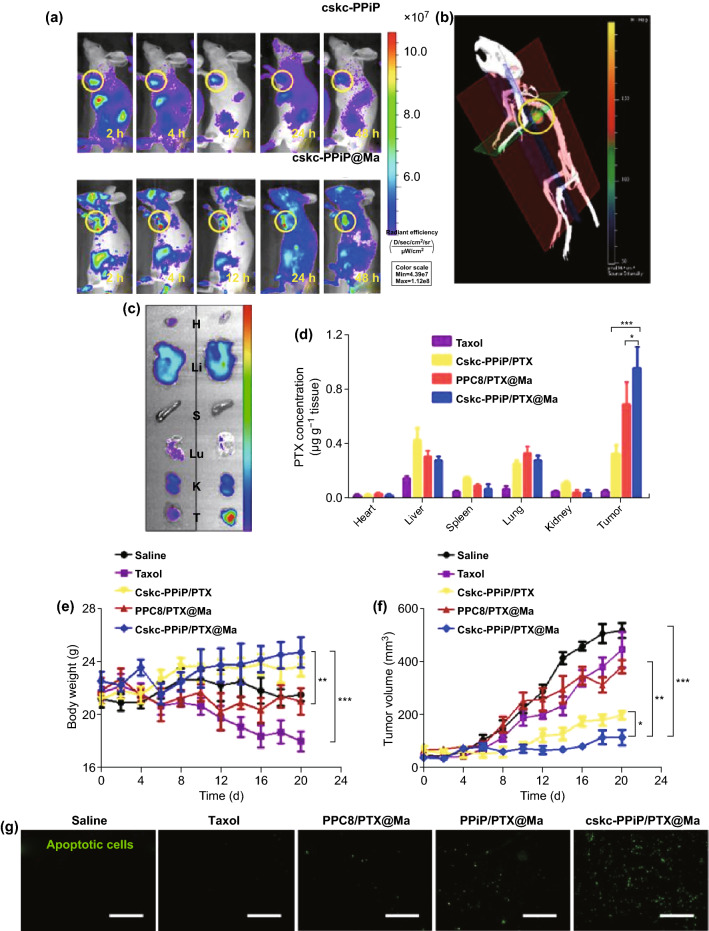
**a** Mice were injected with either cskc-PPiP or cskc-PPiP@Ma NPs loaded with an IR probe for the indicated amount of time. **b** 3D reconstructed fluorescent image of signal in a mouse 48 h following cskc-PPiP@Ma treatment. **c** Heart (H), liver (Li), spleen (S), lung (Lu), kidney (K), and tumor (T) tissues isolated from the mouse in **b**. **d** PTX levels in the organs of mice following treatment using PTX, cskc-PPiP/PTX, PPC8/PTX@Ma, or cskc-PPiP/PTX@Ma (*n* = 4). **e** Body weight and **f** tumor volumes over 3 weeks in treated mice. **g** Tumor tissues from mice treated as indicated were assessed for apoptotic cells, shown in green. Scale bar = 100 μm. Adapted from Ref. [126] with permission

There remains a need for more rational efforts to develop membrane-coated NPs suited for both efficient drug delivery and release in target tissues. In one recent study, macrophage membrane-coated cskc-PPiP/PTX@ Ma NPs were generated as an approach to efficiently targeting drugs to tumor sites wherein they are gradually released in response to local microenvironmental changes in tumor pH [126]. Once these particles arrive in the tumor site, the microenvironment promotes shedding of the macrophage membrane, allowing the released surface-modified NPs to effectively penetrate and deliver drug to the tumor directly. For this study, authors functionalized a pH-sensitive polymer using the cationic 2-aminoethyldiisopropyl group (PPiP), thereby adjusting its pH buffering potential to match the extracellular tumor environment. They also generated the synthetic D-form cskc oligopeptide to mediate NP targeting, with PTX being used as a model for drug delivery in an orthotopic mouse model of breast cancer. The authors were able to clearly demonstrate effective accumulation of macrophage-coated particles in tumors via fluorescent imaging and 3D reconstructions (Fig. 9a, b), with similar biodistribution in the tumor and in key organs (the heart, liver, spleen, lung, and kidney) (Fig. 9c). Owing to its ability to mediate tumor targeting, the PTX-loaded coated NPs bearing the cskc motif were, in contrast, preferentially enriched in the tumor (Fig. 9d), resulting in substantial tumor control without any corresponding decrease in overall body weight (Fig. 9e, f). There was also clear evidence of widespread tumor cell apoptosis in mice treated with these cskc-PPiP/PTX@Ma particles (Fig. 9g).

There is an ever-present need for strategies that can improve the efficacy of chemo-radiotherapy while reducing associated site effects. To this end, Ju et al. [127] developed an approach to neoadjuvant therapy in which they combined human neutrophils with Abraxane-loaded cytopharmaceuticals and radiotherapy as a means of treating gastric cancer. They utilized peripheral blood neutrophils to internalize Abraxane, which is a PTX-loaded NP, yielding a cytopharmaceutical agent. Localized tumor irradiation can both directly kill tumor cells and induce the expression of inflammatory IL-8, IL-10, and TNF-α for at least 48 h, with the later cytokines promoting the recruitment of the neutrophil cytopharmaceuticals to tumors, wherein they are activated to release both neutrophil extracellular traps (NETs) and Abraxane, thereby providing a dual approach to tumor cell killing.

WBC-coated NPs offer an attractive approach to avoiding NP immune detection while facilitating sustained circulation and drug release. Given that there are many different forms of leukocytes available, these cells can be leveraged for a range of distinct targeted drug delivery applications without significant modifications. Given recent advancements in the development of WBC-NPs capable of gradual drug-release in response to the tumor microenvironment, it is clear that this technology is steadily progressing towards more intelligent therapeutic strategies.

### Phototherapy

**Fig. 10:**
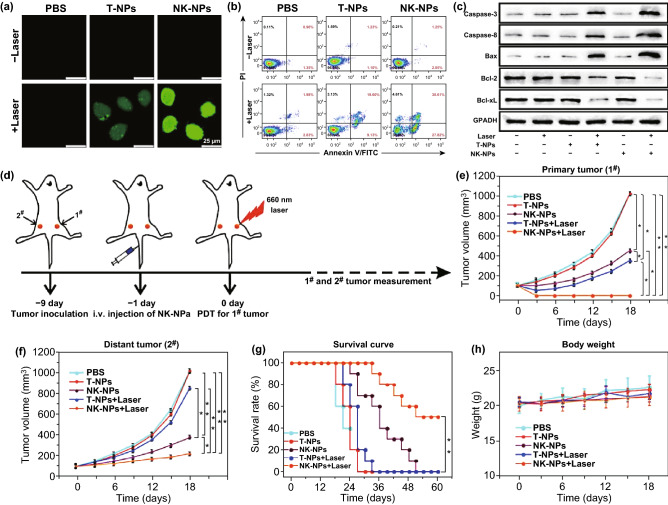
**a** ROS production in cells exposed to T- or NK-membrane NPs following 660 nm irradiation (100 mW cm^−2^) was assessed using the fluorescent DCFH-DA indicator. **b** Flow cytometric assessment of apoptotic induction in irradiated cells exposed to T- and NK-NPs. **c** Western blotting-mediated measurement of apoptosis-associated proteins in response to NK-NP + PDT treatment. **d** Overview of the study experimental design, with a dual 4T1 tumor implant model in which primary tumors on the right side received PDT, whereas distal tumors on the left side did not. **e** Primary tumor growth. **f** Distal tumor growth. **g** Morbidity-free survival of differently treated mice. **h** Changes in body weight of differently treated mice. (*n* = 10). (*p < 0.05, **p < 0.01). Adapted from Ref. [128] with permission

The membranes of NK cells are able to induce M1-polarization of macrophages in order to achieve cell-membrane-mediated immunotherapy. This is of particular value given that PDT approaches are often coupled with efforts to induce immune responses in the context of anti-cancer therapies. As such, Deng et al. [128] developed NK cell membrane-coated NPs (NK-NPs) loaded with 4,4′,4″,4‴-(porphine-5,10,15,20-tetrayl) tetrakis (benzoic acid) (TCPP). When the NK-NPs were i.v. injected into mice that were then irradiated with an appropriate light source, they observed NK membrane-mediated killing of cells in a manner consistent with responses for human NK cell membranes (Fig. 10a–c). To test whether the NK-NPs were more effective than other therapeutic strategies and better suited to treating pre-existing tumors, the authors employed a bilateral 4T1 tumor implant model (Fig. 10d). In this system, they found that the NK-NPs were, in combination with PDT, able to eliminate primary tumors through a synergistic mechanism (Fig. 10e, f). The NK-NPs were also able to slow the growth of distal tumors away from the site of PDT in an abscopal manner, and they found that half the mice in the NK-NP + PDT group survived for 60 days over the course of the study period (Fig. 10g). This treatment was not associated with any significant changes in body weight relative to control mice, indicating good therapeutic tolerance (Fig. 10h).

Similarly, WBC membranes can be employed to enhance PTT efficacy. For example, macrophage membrane-coated gold nanoshells (MPCM-AuNSs) have been devised as a novel PTT agent useful for in vivo cancer therapy [129]. In this study, the authors utilized a 4T1 tumor model to demonstrate that macrophage membrane-coating of the nanoshells improved their biocompatibility and tumor targeting ability while extending their time in circulation to over 48 h. When used to treat mice via a PTT approach, these MPCM-AuNSs allowed for effective tumor growth inhibition following NIR irradiation, with a near complete elimination of tumors within a 25-day study period. In a similar approach, another group developed macrophage membrane-coated iron oxide (Fe_3_O_4_) photothermal NPs [130]. These particles exhibited excellent biocompatibility, tumor targeting, and immune evasion, and when i.v. injected into MCF-7 tumor model mice these particles led to a marked increase in tumor temperature from 34.4 to 55.6 °C within 5 min of irradiation, leading to clear tumor regression.

By accurately targeting NPs for PTT, the overall efficacy of this approach can be markedly improved. One recent study therefore utilized gold-silver nanocages encapsulated in a macrophage membrane that was first bacterially pre-treated, thereby allowing for more efficient bacterial targeting [131]. When these resultant NPs were coupled with localized NIR irradiation at the site of infection, the temperature at this site rapidly rose to 50.9 °C, mediating efficient bacterial destruction.

PTT efficacy can also be enhanced by rationally combining this approach with specific therapeutic compounds. For example, Zhao et al. [132] designed Bi_2_Se_3_ NPs coated in a macrophage membrane and loaded with quercetin that were able to release the chemokine CCL_2_ in response to hyperthermic conditions, thereby mediating cellular recruitment and impairing breast cancer growth and metastasis. When used in mice bearing 4T1 tumors, in vivo imaging revealed that these coated particles accumulated within the tumor within 4 h of i.v. injection, and were able to remain there for as long as 24 h. Upon appropriate NIR irradiation, local temperatures rose as high as 70 °C. When particles were also loaded with quercetin, there was a clear decrease in tumor volume following irradiation, demonstrating clear PTT efficacy. In another recent strategy, researchers have utilized cytopharmaceuticals to mediate a combination of PTT and inflammation-mediated active targeting (IMAT) chemotherapy, first conducting PTT 72 h after injecting animals with PEGylated gold nanorods, and then administering cytopharmaceutical agents to mediate IMAT chemotherapy [133]. This dual treatment approach led to localized inflammation in the tumor, with the produced inflammatory factors mediating neutrophil recruitment and more effective tumor clearance.

WBC-NPs have thus been shown to be ideal agents well-suited to PDT and PTT approaches, improving the biocompatibility and targeting potential of active photosensitization/photothermal compounds in vivo. These leukocyte membranes offer an effective means of ensuring that NPs are targeted specifically to sites of tumors or infections to a greater extent than uncoated NPs.

### Immune Modulation

Beyond the above approaches, the biomimetic potential of WBC-NPs has also led to interest in their use for immunomodulatory therapies. For example, Zhang et al. [54] have developed CD4^+^ T cell-coated NPs with a polymeric core (T-NPs) which they were able to target HIV viral particles. Specifically, as the T-NPs expressed CCR5 and CXCR4, which are T cell co-receptors necessary to bind to HIV, the T-NPs were able to selectively bind the HIV gp120 glycoprotein and to disrupt the resultant gp120-mediated killing of proximal CD4^+^ T cells. The T-NPs could also inhibit HIV infection of human PBMCs and monocyte-derived macrophages in a dose-dependent fashion.

**Fig. 11:**
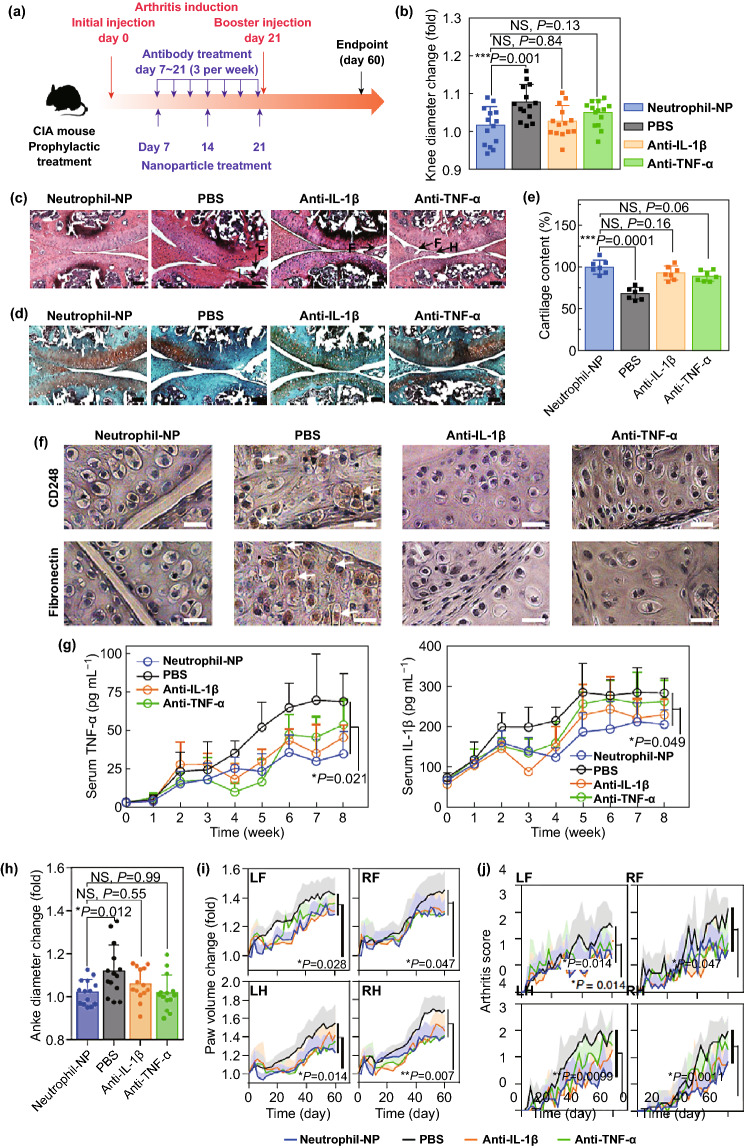
Neutrophil-NPs reduce joint destruction and elicit a systemic therapeutic response following a prophylactic regimen. **a** Overview of the prophylactic regimen used in a CIA mouse model. **b** Changes in hind knee diameter over 60 days following CIA induction relative to day 0. **c**, **d** Representative H&E and safranin-O staining of knee sections prepared from mice treated using PBS, neutrophil-NPs, anti-IL-1β, or anti-TNF-α. Scale bar = 100 μm. F, synovial membrane fibrillation; H, synovium hyperplasia; I, immune infiltration. **e** Quantification of cartilage levels in differently treated mice as measured in safranin-O-stained sections. **f** Representative CD248 (upper) and fibronectin (lower) staining of knee sections from differently treated mice. Scale bar = 10 μm. **g** Concentration of TNF-α and IL-1β in the serum of CIA mice in different groups. **h** Changes in hind ankle diameter on day 60 after arthritis induction compared to that on day 0. **i**, **j** Values of paw volume and arthritis score were recorded every other day for a total of 60 days. All data points represent mean ± S.D. (*n* = 7 CIA mice). *p ≤ 0.05, **p ≤ 0.01, ***p ≤ 0.001. One-way ANOVA with Dunnett’s post hoc analysis. Adapted from Ref. [134] with permission

In another study, Zhang et al. [134] demonstrated that rheumatoid arthritis (RA), an autoimmune-mediated inflammatory disease of the joints, could be treated using NP-mediated delivery of broad-spectrum anti-inflammatory compounds. In this study, the authors employed neutrophil-coated NPs in a murine collagen-induced arthritis (CIA) model in order to explore anti-arthritic activity (Fig. 11a). At the end of the study period, the authors found that mice treated with neutrophil-NPs had smaller knee diameters than control PBS-treated mice consistent with better disease control, with responses being comparable to traditional anti-inflammatory treatments (anti-IL-1β and anti-TNF-α) (Fig. 11b). Consistent with this, neutrophil-NPs were better able to reduce immune infiltration of the cartilage and consequent cartilage degradation as demonstrated via histological examination (Fig. 11c-e). The majority of FLS in the control group were CD248^+^ and fibronectin^+^, whereas those in mice treated with neutrophil-NPs or anti-inflammatory antibodies were negative for these markers (Fig. 11f). The authors also examined systemic inflammation in these CIA model animals, measuring circulating TNF-α and IL-1β levels, as both are linked to arthritic disease severity. They found levels of both cytokines to be reduced in animals treated with anti-cytokine antibodies or neutrophil-NPs, consistent with effective systemic disease control (Fig. 11g). Importantly, in neutrophil-NP treated mice, knee and ankle joint diameters were the lowest of all treated animals (Fig. 11h), and paw swelling was markedly reduced by neutrophil-NPs and anti-cytokine treatments relative to control PBS-treated mice (Fig. 11i). The authors further found that neutrophil-NPs were able to effectively reduce arthritic severity better than PBS when mice were assessed in a blinded manner (Fig. 11j), thus confirming their therapeutic efficacy.

Given the unique properties of different forms of leukocyte membranes, there remains a wide array of possible applications for WBC-NPs in the treatment of immunological diseases. These examples of T cell membrane-mediated HIV targeting and neutrophil-mediated cartilage targeting to protect joints via microbubble production offer clear a valuable therapeutic opportunity, with future research efforts likely to develop further exciting and novel immunomodulatory interventions.

In summary, different subtypes of leukocytes perform different functions, and they also differ in practical applications. Macrophages are often used in cargo delivery because of their long circulation in vivo and their ability to mediate immune evasion through self-recognition by other macrophages. Lymphocytes, such as T cells, B cells, and NK cells, are more widely used in targeted delivery, especially in cancer therapy. Notably, unlike T and B cells, NK cells can directly target cancer cells through interaction with inhibiting and activating receptors on cancer cell surface. As the most abundant WBCs in peripheral blood, neutrophils play a key role in combination with chemotherapy or radiotherapy due to their natural chemotaxis to inflammatory signals.

## Cancer Cell Membrane-Coated Nanoparticles

Cancer cells represent another potentially viable source of membrane material for NPs coating, and are of particular interest owing to the fact that many cancer cells are able to effectively undergo homologous adhesion to other cancer cells [135]. Specific adhesion proteins on the surface of the cancer cell types can mediate their effective self-recognition, allowing for homing to homologous tumor sites and thus enabling NPs coated in such membranes to effectively target cancer cells even when other heterologous tumor cells are also present [136–138]. Cancer cell membrane-coated NPs can also be designed so as to possess a high degree of stability while also bearing normal cancer cell membrane antigens, allowing for effective delivery of both multivalent tumor antigens and immunostimulatory adjuvants to tumor sites and thus improving the efficacy of cancer vaccination efforts via inducing tumor-specific immunity [139].

### Drug Delivery

**Fig. 12:**
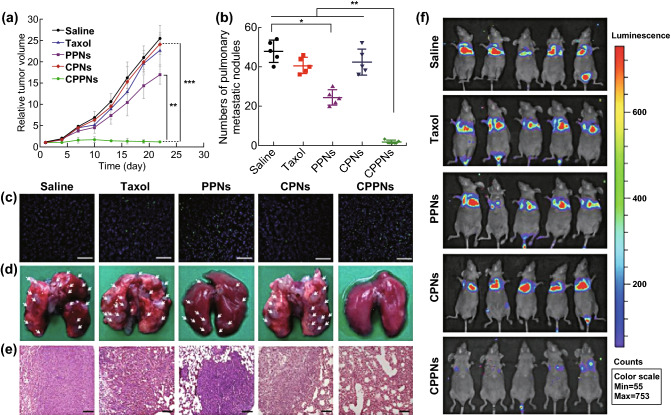
**a**–**e** In vivo effects of CPPNs in mice implanted with 4T1 tumors. **a** Tumor-growth and **b** lung metastases in differently treated mice. **c** Tumor TUNEL staining. Scale bar = 200 μm. **d** Lung tissues and **e** H&E stained lung tissues following experimental termination. Scale bar = 100 μm. White arrows indicate metastases. **f** *In vivo* bioluminescent imaging of mice in an i.v. 4T1 metastatic model. Data are mean ± SD (*n* = 5). Statistical significance: *p < 0.05, **p < 0.005, and ***p < 0.0005. Adapted from Ref. [141] with permission

Multifunctional nanocarrier-based treatment is aimed at overcoming certain key challenges in cancer therapy [140]. While traditional chemotherapeutic drugs lack effective tumor targeting capabilities, coating the drugs in cancer cell membranes can improve targeting efficacy. For example, Li et al. [141] developed a novel cancer cell-biomimetic NP loaded with PTX that they were utilized for targeted chemotherapy in a 4T1 tumor model system. The resultant cancer-cell-membrane-coated PPNs (CPPNs) retained normal expression of 4T1 surface antigens such as E-cadherin, CD47, and TF antigen, and were able to effectively accumulate in primary tumors and metastases when injected into mice implanted with homotypic 4T1 tumors. The CPPNs mediated effective tumor growth inhibition when used to treat these mice (Fig. 12a), reducing tumor volumes to 4.8% of those in the control group, as compared with a reduction to only 66.4% for uncoated PPNs, thus demonstrating the clear efficacy of cancer membrane-coated NPs. Consistent with this, CPPNs induced higher rates of tumor apoptosis than other treatments (Fig. 12c), and were associated with fewer lung metastases than control PBS treatment (Fig. 12b, d), reducing rates of metastasis by 97.8% as confirmed by lung H&E staining (Fig. 12e). This was also confirmed via in vivo bioluminescent imaging (Fig. 12f), thus indicating that CPPNs can improve drug delivery efficiency and therapeutic PTX efficacy.

Cancer cell membranes can also be employed for functional nanoreactor development. Balasubramanian et al. [142] used undecylenic acid-modified thermally hydrocarbonized PSi (UnPSi) NPs surrounded by an MDA-MB-231 breast cancer cell membrane to encapsulate the horseradish peroxidase (HRP) enzyme. The surrounding membrane served as a layer that was able to allow chemicals to travel in or out of the cell while retaining the HRP enzyme inside, thus producing an effective nanoreactor wherein HRP was able to effectively reduce intracellular ROS levels. In another approach, a group developed a CaCO_3_-capped MSN in order to allow for controlled drug release in tumors in response to the local acidic microenvironment [143]. For this approach, MSNs were loaded with DOX and surrounded by a CaCO_3_ layer, followed by external coating with membranes derived from LNCaP-AI cells. The authors found that in a normal simulated physiological setting, drug release from these particles was negligible, whereas in an acidic setting mimicking the tumor microenvironment DOX was rapidly released. Importantly, the ability of cancer cell membranes to mediate homotypic targeting allowed the particles to be readily targeted to model prostate tumor cell sites, thereby achieving a more pronounced anti-tumor effect.

The combination of DOX and iron oxide compounds enclosed in cancer cell membranes has been shown being an efficacious approach to cancer cell drug delivery and treatment. Zhu et al. [137], for example, utilized an iron oxide nanoplatform loaded with DOX and coated in cancer cell membranes and found the resultant NPs effectively targeted to homotypic tumors in a murine dual tumor model. By using mice bearing two different tumor types (H22 and UM-SCC-7) on opposite flanks and then administering mice H22 membrane-coated NPs, the authors demonstrated homotypic targeting to H22 tumors with minimal NP accumulation in the heterologous UM-SCC-7 tumors. When loaded with drugs, the NPs allowed for potent in vivo tumor treatment. In a similar approach, DOX-loaded iron oxide NPs coated in human squamous carcinoma membranes were also able to mediate effective homotypic cancer cell targeting [144]. In this model, NPs coated in UM-SCC-7 membranes failed to efficiently interact with the heterologous COS7 tumor cells, thereby effectively inhibiting the growth of UM-SCC-7 but not COS7 tumors in this model.

Nanocarrier targeting efforts often rely on complex bottom-up strategies. The use of cancer cell membranes to achieve homologous tumor targeting in a top-down fashion represents a novel and appealing approach to efficiently and effectively targeting tumors in patients.

### Phototherapy

In addition to its clear utility in the context of anti-tumor therapy, the coating of NPs in cancer cell membranes has also been used for effective phototherapeutic interventions. Li et al. [49] found that tumor PDT could be effectively mediate by using a tumor targeting nanoplatform containing tirapazamine in a PCN-224 porphyrinic metal organic framework and coated with a 4T1 tumor cell membrane. The resultant NPs could achieve effective immune evasion and 4T1 tumor cell targeting. Once taken up by 4T1 tumor cells, the PCN-224 in the NPs produces high levels of ROS upon irradiation (660 nm, 200 mW cm^−2^, 10 min), mediating effective PDT cytotoxicity. The subsequent hypoxia induced by oxygen utilization then offers an opportunity for tirapazamine activation, resulting in improved chemotherapeutic efficacy. In this study, the 4T1-coated NPs loaded with tirapazamine displayed the strongest efficacy of all tested treatments owing to their ability to durably accumulate in tumor sites.

**Fig. 13:**
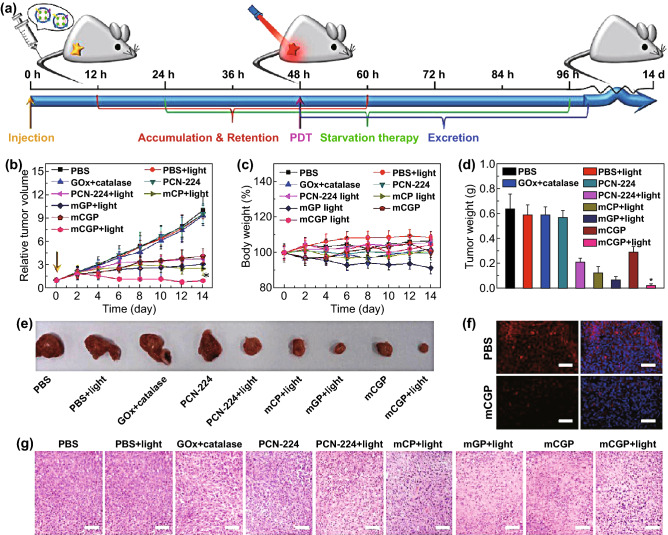
In vivo mCGP anti-tumor efficacy. **a** Overview schematic of the approach to combination PDT/tumor starvation achieved via mCGPs. **b** Tumor volumes and **c** changes in body weight 14 days following indicated treatments, *p < 0.05. Arrows correspond to therapeutic administration. **d** Average tumor weight and **e** representative images following 14 days of indicated treatments. *p < 0.05. **f** Immunofluorescent HIF-1a staining following PBS or mCGP treatment. **g** H&E stained tumor sections on day 14 in differently treated mice. Scale bar = 200 μm. Adapted from Ref. [145] with permission

Li et al. [145] also developed a bioreactor in which glucose oxidase (GOx) and catalase were enclosed in a PCN-224 framework that was in turn surrounded by a 4T1 tumor membrane as above. The authors demonstrated that following administration, the particles (termed mCGPs) accumulated in the tumors of mice bearing 4T1 tumors within 12 h of injection, reaching peak accumulation within 48 h (Fig. 13a). Following irradiation (660 nm, 29.8 mW cm^−2^, 5 min) at this 48 h timepoint, the authors achieved simultaneous PDT and cancer starvation, resulting in synergistic suppression of tumor growth for 14 days following treatment (Fig. 13b). Importantly, decreased HIF-1α immunofluorescence at tumor sites in treated mice were observed, indicating that the mCGPs could mediate tumor H_2_O_2_ breakdown to effectively overcome the hypoxic nature of the tumor microenvironment (Fig. 13c). Tumor weights (Fig. 13d) and images (Fig. 13e) also confirmed the efficacy of mCGP treatment, with growth inhibition rates of 97.1% in irradiated and mCGP-treated mice. H&E staining results further confirmed that this combination strategy was highly effective for mediating cancer cell death (Fig. 13g).

In one recent study, researchers made efforts to develop cancer cell membrane-coated NPs suited to be used in synergistic PDT and cancer starvation therapy [146]. The researchers generated cancer membrane-coated mesoporous NPs to bear both surface GOx and the photosensitizer chlorin e6 (Ce6), and then used the modified NPs to encapsulate bis-[2,4,5-trichloro-6-(pentyloxycarbonyl)phenyl] oxalate and perfluorohexane. The resultant nanoreactor particles could be readily targeted to homologous tumors and were able to evade immune detection, with PFC delivering oxygen to the tumor and thus altering the hypoxic nature of the local microenvironment, and leading to higher rates of glucose and ROS production. In a murine B16F10 lung metastasis model, the nanoreactor particles could completely eliminate lung metastases in vivo.

Researchers also developed a persistent biomimetic luminescent nanoplatform suitable for tracking and irradiation-mediated chemotherapy and PDT treatment of metastatic tumors [147]. To develop this system, a silicon phthalocyanine (Si-Pc) functionalized nanoplatform PLNP core (SPLNP) was developed and surrounded with a silicon-based layer loaded with DOX and an outer cancer cell membrane. The external CCM layer prevented DOX leakage in the bloodstream and mediated targeting to homotypic metastatic tumor cells. The particles were also readily trackable in vivo as a consequence of the “afterglow” effect of NIR irradiation of SPLNPs, allowing for sustained reactive oxygen singlet generation that facilitates rapid nanoplatform endosomal escape and drug release. The net effect of these features was a strategy allowing for combination PDT and chemotherapeutic targeting of tumor metastases, completely (> 99%) inhibiting 4T1 tumor growth in vivo following particle injection and irradiation as compared to control PBS-treated mice.

NPs coated in cancer cell membranes have also been successfully employed as an optimal PTT therapeutic.For example, Chen et al. [148] employed CCM-NPs loaded with ICG (termed ICNPs) as a means of conducting targeted PTT in mice. Using an MCF-7 tumor model, the investigators administered either uncoated or coated ICGs to mice and then irradiated animals with an appropriate laser (1 W cm^−2^, 5 min), resulting in a temperature rise to 48.2 °C for the uncoated particles and an increase to 55.3 °C for the coated particles, owing to their enhanced homologous tumor targeting abilities. On day 18, mice administered the coated ICNPs and irradiated exhibited complete tumor remission, without incidence of relapse and with a 100% survival rate.

**Fig. 14:**
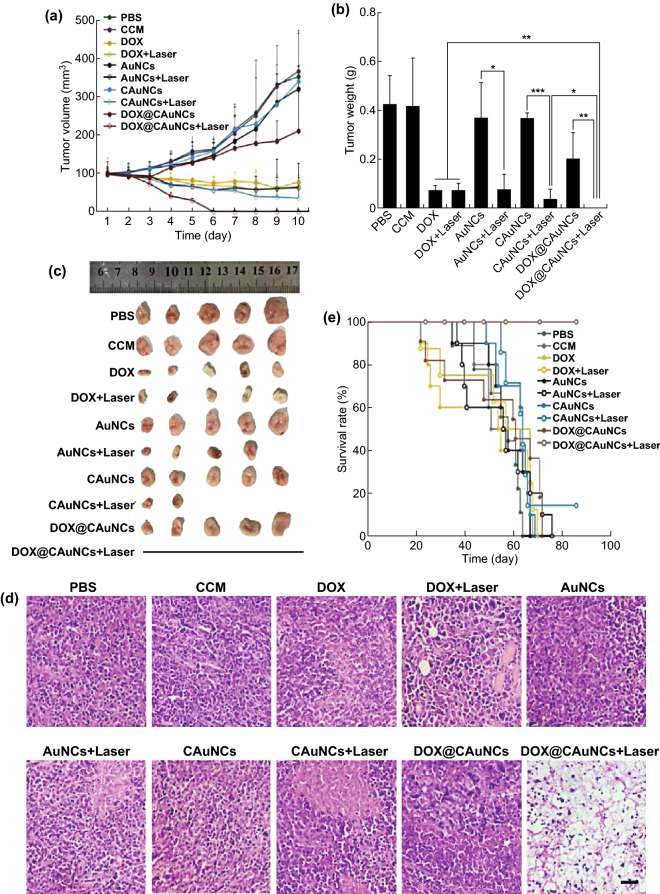
DOX-loaded CAuNCs mediate synergistic PTT and chemotherapy. **a** H22 tumor growth in mice following treatment with the indicated Au/DOX doses (10 and 15 mg kg^−1^) with or without irradiation (10 min; 808 nm; 1 W cm^−2^) once daily for 4 days (*n* = 13). **B** Tumor weight after treatment (*n* = 5). **c** Photos of tumors after treatment. **d** H&E stained tumor sections from mice treated as in **a**. Scale bar = 50 μm. **e** Kaplan–Meier survival curve for mice treated as in **a** (*n* = 8). Data are mean ± SD, *p < 0.05, **p < 0.01, ***p < 0.001. Adapted from Ref. [40] with permission

H22 CCM-coated gold nanocages (CAuNCs) loaded with DOX was developed by using an ammonium sulfate gradient approach [40]. The particles were administered in vivo, and were found to efficiently accumulate in tumors to a roughly twofold greater extent than did uncoated AuNCs. When mice were administered the CAuNCs and then irradiated with an 808 nm NIR laser after 24 h and once daily for 4 days, DOX was efficiently released from the particles in the tumor site (Fig. 14a-c). Both AuNCs and CAuNCs alone were able to achieve therapeutic efficacy upon irradiation, but only DOX-loaded CAuNCs were able to achieve complete tumor eradication in the mouse model, highlighting their potent ability to mediate combination PTT-chemotherapy. These observations were consistent with histological findings and demonstrated more significant structural damage in the tumors of mice administered DOX-loaded CAuNCs following irradiation (Fig. 14d), with comparable survival findings (Fig. 14e).

Yet another group generated carrier-free HeLa CCM-NPs containing both DOX and ICG, achieving an 89.3% drug loading rate while maintaining both immune evasion and homologous cancer cell targeting capabilities [149]. Owing to the photothermal properties of ICG, in response to NIR irradiation (808 nm, 3 W cm^−2^) the membrane of the particles was disrupted, allowing for efficient drug release. This coupled with the EPR-mediated homologous targeting ability of the particles, resulting in both high intratumoral accumulation and temperature elevation to 52.4 °C upon irradiation. When employed in mice bearing HeLa tumors, administration of the NPs followed by irradiation mediated robust anti-tumor efficacy, markedly shrinking tumors in treated animals.

In summary, several rational and efficacious approaches to CCM-NPs have been developed for phototherapeutic applications. These particles are able to effectively target homologous tumors to deliver photosensitizers/photothermal compounds, and in combination with chemotherapy or tumor starvation strategies these particles can achieve marked anti-tumor efficacy.

### Immune Modulation

CCM-NPs also represent an effective means of inducing immune responses specific to cancer cells. Jin et al. [57] coated PLGA NPs with CCMs, using membranes derived from CXCR4-high or –low and CD44-high or –low glioma and breast cancer cells. They demonstrated specific increases in CD8^+^ T cell responses following NP treatment when membranes were derived from CXCR4-high U87 glioma cells. The mice also had higher levels of CD4^+^ T cells in the spleen, and the highest frequency of interferon gamma (IFNγ) producing T cells as measured by ELISpot assay.

Zhang et al. [139] generated NPs coated with both the TLR4 agonist monophosphoryllipid A (MPLA) as an adjuvant and an outer CCM layer that were suitable for use in a vaccinal context. In the experiments, they generated CCM-NPs using B16F10 tumor cell membranes, and then used these cells to assess dendritic cell maturation and tumor antigen delivery. Only by combining the MPLA adjuvant layer with the CCM coating were the authors able to achieve effective dendritic cell (DC) maturation, as assessed based on increased CD40, CD80, and CD86 expression. They then combined the DCs with transgenic pmel-1 murine splenocytes specific for a particular tumor antigen (a glycoprotein 100 epitope), in order to assess how the NPs promoted DC tumor antigen presentation. The authors observed significant clustering of T cells around CCM and MPLA-coated NP-pulsed DCs and a significant induction of IFNγ secretion, consistent with a CCM-MPLA-NP-mediated activation of antigen-specific cytotoxic T cell responses.

In another cancer vaccination approach, Yang et al. [33] generated adjuvant-containing NPs with a mannose-modified external CCM layer. The authors loaded PLGA NPs with the TLR7 agonist imiquimod (R837), with the external CCM layer serving as a source of tumor antigens. By modifying the external surface of the particles with mannose residues, the authors were able to enhance uptake of their nanovaccine by antigen presenting DCs, thereby offering a novel approach to enhance the development of specific anti-tumor immune responses. The nanovaccine particles were effective when administered prophylactically to mice, and were also effective when used therapeutically to impair myeloma progression when used in combination with the administration of anti-PD-1.

Another group fabricated a NP-based adjuvant-containing tumor vaccine by loading PLGA NPs with the TLR agonist CpG and then coating the NPs in CCM derived from B16F10 melanoma cells [150]. The resultant NPs, termed CpG-CCNPs, were then used to promote antigen-specific T cell responses, with mice vaccinated using CpG-CCNPs protected from tumor development in 86% of cases over a 150-day study period. When the CpG-CCNPs were combined with checkpoint blockade antibodies (anti-CTLA4 and anti-PD1), control of established B16F10 tumor growth was markedly improved, with median survival increasing from 18 to 32 days relative to control animals. The results of this study clearly demonstrated that adjuvant-loaded CCM-NPs are viable as both tumor vaccines and as therapeutic agents for cancer treatment in combination with other immunotherapeutic agents.

In another example of CCM-NPs used for cancer immunotherapy, Fontana et al. generated thermally oxidized porous silicon (TOPSi) NPs encapsulated in AcDEX or spermine-modified AcDEX (SpAcDEX) polymeric particles. Through co-extrusion, these particles were coated with MDA-MB-231 breast cancer CCM vesicles, and then functionalized with the Trp2 model antigen. The resultant NPs were highly cytocompatible in immortalized human cell lines (KG1 and BDCM), and could significantly enhance IFN-γ secretion from human PBMCs without inducing IL-4 production, thus specifically promoting the Th1 polarization of newly primed T cells.

Cancer cell membranes offer a full array of tumor-associated antigens to stimulate robust tumor-specific immune responses. The above examples have confirmed that encapsulating vaccine adjuvants in cancer cell membranes is an efficient method for stimulating anticancer immunity, offering tremendous potential for cancer immunotherapy.

## Other Cell Membrane-Coated Nanoparticles

### Stem Cell Membrane-Coated Nanoparticles

Mesenchymal stem cells (MSCs) can be readily isolated and can undergo long term in vitro proliferation. These MSCs also possess properties such as long-term circulatory potential, immune evasion, and tumor targeting properties which make them ideal for NP delivery [23]. The cells express a range of ligands well-suited to tumor targeting, and readily migrate to inflamed tissues in vivo due to this property [17]. Yang et al. [151] coated PLGA NPs using umbilical cord MSC membrane to achieve targeted DOX delivery to MHCC97-H liver tumors in nude BALB/c mice. By functionalizing the MSC membrane, they achieved a high degree of NP uptake and efficient tumor targeting and cancer cell killing, markedly inhibiting tumor growth by 78.2% in vivo. In a similar effort, another group coated gelatin-based nanogels in MSC membrane in a top-down manner to achieve high rates of stability and tumor targeting in vitro and in vivo [50]. The resultant DOX-loaded particles more effectively inhibit the growth of HeLa tumors than were free DOX or uncoated gelatin-DOX particles, thus demonstrating the enhanced therapeutic efficacy associated with MSC membrane coating.

In order to engineer particles which home to ischemic tissues, one group recently developed stem cell membrane-coated nanocarriers functionalized to express CXCR4 [152]. In this strategy, the authors effectively increased particle retention in ischemic tissues via CXCR4 functionalization of the nanocarrier membrane. The researchers relied upon the use of human adipose-derived stem cells overexpressing CXCR4 in order to generate these functionalized membranes for their PLGA nanocarriers. Coating with CXCR4-functionalized stem cell membrane was linked with improved penetration of the endothelial barrier, as well as with decreased uptake by both human (THP-1; 76–24%) and murine (J774; 84–29%) macrophages. Importantly, CXCR4 expression was readily transferred to and maintained upon the resultant bioengineered stem cell membrane-nanocarriers (BSMNCs). To assess the relative value of CXCR4 functionalization of the NPs, the authors used a mouse model of severe hindlimb ischemia to measure the dynamics of NP targeting in vivo. Besides, by using Cy-5.5-labeled SMNCs or BSMNCs, the authors found the later to more effectively be retained in ischemic tissues over a 14-day period, thus confirming that the BSMNCs may represent an effective therapeutic strategy to drug delivery under ischemic conditions.

MSC-coated NPs are also employed in the context of phototherapeutic regimens. For example, a research group used MSC-derived membrane material to effectively camouflage silica-encapsulated β-NaYF4:Yb^3+^, Er^3+^ upconversion NPs [31]. The resultant biomimetic PDT particles were loaded with two photosensitizing agents (ZnPC and MC540) with high efficiency owing to the ideal loading characteristics of the internal particles, and both could be activated by 980 nm irradiation. By using the particles in vitro and in vivo, the authors found that MSC coating could extend circulation, improve tumor targeting, and enhance anti-tumor efficacy. In a separate approach, the researchers used MSC membrane-coated Fe_3_O_4_ polydopamine (PDA) NPs loaded with siRNA molecules. The resultant particles exhibited excellent photothermal efficacy, and were suitable for MR imaging while also allowing for effective siRNA delivery to DU145 cells, with an 84.2% uptake efficiency. By combining both gene silencing and PTT, the authors demonstrated clear in vivo efficacy in the DU145 xenograft mouse model system.

As these results shown, MSCs offer desirable characteristics including extended circulation and tumor targeting, making them ideal for delivery of chemotherapeutic or photosensitizing agents to tumors. By functionalizing the MSC membranes, it is possible to generate even more efficacious NPs capable of readily crossing the endothelial barrier.

### Fibroblast Cell Membrane-Coated Nanoparticles

Activated fibroblasts (AFs) are a major component of the tumor stroma, and have been shown to drive angiogenesis, metastasis, and cancer cell proliferation [153]. Li et al. [154] recently sought to use utilize fibroblast membranes to coat NPs in an effort to mediate tumor-associated AF targeting, thereby allowing for improved multimodal imaging guided cancer therapy. For this approach, the authors utilized semiconducting polymer NPs (SPNs) surrounded by an AF membrane, with the homologous targeting potential of the AF-SPNs allowing them to target cancer-associated AFs. The resultant particles were then used for PDT in vivo in a 4T1 xenograft tumor model, irradiating mice 48 h after particle injection (808 nm; 5 min). This intervention was linked to a maximal temperature increase to 50 °C in mice injected with AF-SPNs, and this increase was 14.0 °C higher than that in control PBS-treated mice. PDT-induced damage was then assessed by staining for sulfenic acids, which are indicative of protein oxidation, and the resultant images confirmed higher levels of damage-associated green fluorescence in the tissues of irradiated mice administered these SPNs. Importantly, the fluorescent signal was stronger for mice administered AF-SPNs than for mice administered uncoated SPNs, and fluorescence was largely absent in PBS control animals. Irradiation of AF-SPN mice was associated with superior inhibition of tumor growth relative to other treatment groups, leading to complete PDT-mediated tumor ablation.

Another recent strategy has relied upon the use of fibroblast membrane-coated NPs for diabetes treatment. For example, Tan et al. [155] coated a PLGA diaphragm with fibroblast membrane, and were then able to differentiate pancreatic stem cells on the surface to promote the generation of insulin-secreting cells. This approach offers an opportunity to increase PLGA membrane biocompatibility while effectively controlling the differentiation of pancreatic stem cells, indicating that such a functionalized PLGA surface may be useful for the generation of artificial islets that can be used to treat individuals suffering from diabetes.

### Beta Cell Membrane-Coated Nanoparticles

Pancreatic beta cells are an endocrine cell type that make up 70% of pancreatic cells, and are found in islets. The cells are heavily dependent upon interactions with other cells for survival and functionality, and this property has led investigators to assess optimal scaffold designs with the potential to drive optimal beta cell functionality [156]. For example, Chen et al. generated nanofibers coated in beta cell membrane for beta cell culture. The result showed a marked increase in beta cell survival and proliferation over a 1-week period (with a 327% increase relative to baseline). The resultant cells could also mediate a five-fold increase in glucose-dependent insulin production relative to beta cells grown on nanofibers that had not been modified with beta cell membranes.

### Bacterial Membrane-Coated Nanoparticles

Given their high levels of immunogenic proteins and adjuvants, bacterial cell membranes represent a novel and potentially optimal source of cellular membrane material useful for coating NPs in contexts where pathogen-associated molecular pattern-mediated innate and adaptive immune system activation is desirable [157, 158]. Zhang’s group coated 30 nm gold NPs using membranes derived from *E. coli* [157]. When mice received a subcutaneous dose of the bacterial membrane NPs (termed BM-AuNPs), the authors demonstrated that the particles were trafficked to proximal draining lymph nodes wherein they effectively activated DCs as evidenced by both higher overall CD11c + DC levels and higher frequencies of CD40/CD80/CD86-positivity among DCs in the lymph nodes, suggesting both effective recruitment and activation. When the authors further explored specific B cell-mediated immunity against the bacteria by measuring antibodies specific for *E. coli*, they found that mice vaccinated with the BM-AuNPs exhibited higher avidity antibody than did mice vaccinated with vesicles composed of only the outer membrane vesicles (OMVs). They further examined *E. coli*-specific T cell responses in this experimental system, and determined that both BM-AuNPs and OMVs significantly enhanced IFN-γ and IL-17 production upon infection relative to naive mice, confirming the development of an *E. coli*-specific T cell response. Importantly, the IFN-γ and IL-17 levels were higher for mice immunized using BM-AuNPs relative to those immunized with OMVs, suggesting the former are more effective for T cell activation.

Another group recently sought to explore the potential use of bacteria membrane-coated NPs for active targeting efforts [58]. The researchers utilized PLGA NPs coated in *S. aureus*-derived extracellular vesicles (EVs), yielding particles that were able to actively traffic to *S. aureus*-infected macrophages in vitro, and to major sites of *S. aureus* infection in vivo in mice. Whereas the particles achieved active targeting, comparable NPs instead coated using either a PEGylated lipid bilayer or *E. coli* OMV failed to exhibit this targeting activity. Consistent with the observed targeting activity, the EV-coated particles more readily accumulated in organs with a higher bacterial burden (the kidney, spleen, lungs, and heart) in infected mice relative to uninfected controls. When the EV-NPs were loaded with antibiotics, they significantly enhanced antibiotic efficacy upon i.v. administration, markedly reducing bacterial burden, particularly in the lung and kidneys.

Recent studies have explored the use of PDT as a strategy for overcoming antibiotic resistance. In one study, authors combined the photosensitizer protoporphyrin IX (PpIX) with the antimicrobial peptide (KLAKLAK)_2_ (KLA), thereby producing PPK particles ideal for bacterial PDT inactivation [159]. The particles readily bound bacterial cells via a combination of electrostatic interactions and membrane insertion, damaging the membrane and driving PpIX-induced ROS-mediated cytotoxicity upon 660 nm light exposure. Importantly, the PPK particles were highly effective at killing both Gram-positive and -negative bacteria (*S. aureus* and *E. coli*, respectively).

### Hybrid Cell Membrane-Coated Nanoparticles

Beyond the use of individual cell membranes, recent work has sought to combine multiple membrane types in order to develop unique hybrid membranes with enhanced functional characteristics that can then be used for novel NP coating and delivery strategies.

A dual-membrane-coated hybrid NPs was developed by combining RBC and platelet membrane material with the properties of both source cell types [46]. The hybrid [RBC-P] NPs could circulate for extended periods of time, making them well-suited to future studies in vivo. Researchers also developed a hybrid RBC/platelet membrane coated polypyrrole (PPy) NPsthat could mediate tumor cell killing upon NIR irradiation [160]. The hybrid RBC-platelet-coated PPyNPs exhibited both RBC and platelet-like attributes, and achieved self-targeting and extended circulation in vivo. To test the functional utility of the particles, mice bearing HCT116 tumors with i.v. injected with the hybrid membrane-coated NPs, after which a NIR laser (808 nm, 1.5 W cm^−2^, 300 s) was used to damage the tumor vasculature, inducing microthrombi formation. The platelet membrane characteristics of the NPs allowed them to readily traffic to the thrombotic areas, yielding superior anti-tumor efficacy than in other treatment groups.

The unique properties of hybrid membranes can also be leveraged in the context of phototherapy to better suppress tumor growth. For example, Wang et al. [161] generated hollow copper sulfide NPs (loaded with DOX) coated with a hybrid of RBC and B16F10 melanoma cell membrane and used as a combination strategy for melanoma treatment. The hybrid particles exhibited characteristics of both RBC and tumor cells, with homologous tumor targeting and extended circulation. Importantly, the particles were also able to completely inhibit melanoma growth when administered to mice. In a similar example, researchers fused membrane materials from RBCs and MCF-7 tumor cells and used this hybrid membrane (RBC-M) to coat melanin NPs [162]. The melanin RBC-M NPs exhibited RBC and MCF-7 cell properties, effectively circulating and targeting MCF-7 cells in vivo. Upon i.v. administration, the authors found that, with a 1:1 RBC:MCF-7 ratio, the melanin RBC-M NPs were most effective, exhibiting superior tumor accumulation and PTT efficacy as compared to uncoated melanin NPs or NPs coated with different cell membrane ratios. For mice treated with the optimal 1:1 melanin RBC-M NPs, following irradiation tumor temperature rapidly rose from 29.6 to 54.0 °C (∆*T* = 24.4 °C) within 10 min, suggesting the particles mediated enhanced PTT relative to uncoated melanin NPs. Consistent with this, the 1:1 melanin RBC-M NPs resulted in the complete elimination of the tumor following PTT in treated animals, with a 100% tumor inhibition rate and H&E- and TUNEL-staining results indicative of enhanced tumor cell destruction, confirming that the hybrid NPs prepared at a 1:1 ratio of RBC to tumor cell material are ideal for PTT therapeutic efficacy in vivo.

As a means of achieving effective targeting to solid tumors, He et al. [163] employed liposomal NPs coated in a hybrid cell membrane composed of both leukocyte and tumor cell material (J774A.1 and HN12 cells, respectively) which were loaded with PTX. When employed in a murine head and neck cancer xenograft model, the authors found that the hybrid membrane-coated NPs exhibited both extended in vivo circulation time and superior targeting to tumors (79.1 ± 6.6% ID per gram of tumor).

Beyond direct cancer treatment, hybrid membrane-coated NPs also have potential in a variety of other specialized applications. For example, Liu et al. [164] recently employed magnetic beads coated in a hybrid WBC and platelet membrane and then surface modified to bear specific antibodies of interest. The resultant hybrid membrane-coated immunomagnetic beads (HM-IMBs) could readily bind cancer cells as a result of their platelet membrane material, and reduce rates of interaction with homologous leukocytes, thereby facilitating rapid and specific circulating tumor cell (CTC) binding. Using blood samples spiked with CTCs, the authors found that coating IMBs with hybrid membrane improved their CTC separation efficiently from 66.68% to 91.77%, while improving the purity of resultant cell preparations from 66.53% to 96.98% as compared with uncoated commercially available IMBs.

In summary, several different research groups have pioneered efforts to combine multiple different types of cell membrane coating strategies, thus greatly expanding the number of options available for biomimetic NP coating efforts. By leveraging the properties of multiple cell types, it is now possible to generate NPs with extended in vivo circulation half-lives and effective targeting abilities, thus offering unprecedented advantages for drug delivery, phototherapy, and CTC separation.

## Application of Cell Membrane-Coated Nanoparticles for Detoxification

Those NPs coated in a membrane could better deliver cargo, facilitate phototherapy, and mediate immunoregulation compared with bare NPs. The proteins present on the surface of the membranes could offer more opportunities to broaden the scope of their utility. Of note, certain exo- and endo-toxic compounds can bind to specific cell surface molecules, and be exploited for detoxification purposes. Indeed, the coated NPs have been explored as a novel strategy for neutralizing specific bacterial toxins owing to the tendency of these toxins to bind the cell membrane [165]. Zhang et al. first proposed such a detoxification approach in 2013. They generated a RBC membrane coated-PLGA NP that served as a “nanosponge” for bacterial toxins in vivo. The coated-NPs could reduce toxicity by preventing them from interacting with their intended targets [166]. The nanosponges could mediate a substantial reduction in staphylococcal alpha-haemolysin (α-toxin)-induced toxicity in vivo, thus representing an ideal method to treat diseases associated with such pore-forming toxins. The RBC nanosponges were also used for the treatment of severe MRSA [167]. The nanosponges could neutralize the hemolytic activity of toxic proteins secreted by the MRSA bacteria in vivo, thus significantly improving murine survival. In addition, when animals were directly challenged by a sublethal dose of supernatant derived from MRSA, the nanosponges significantly decreased the associated lung damage and reduced splenic inflammatory transcription factor activation. The same general detoxification mechanism can also be employed in the context of 3D bioprinting. For example, Zhang’s group prepared a 3D printed RBC-NP/hydrogel that was ideally suited to detoxification. The material could absorb and thereby neutralize the activity of a wide range of toxins while still permitting blood to flow through in vivo [168].

NPs coated in platelet membranes can also be used as a means of binding and thereby clearing pathological antibodies from systemic circulation. This strategy is of value in the context of certain diseases, such as in immune thrombocytopenia purpura (ITP) wherein autoantibodies mediate pathological platelet destruction. Platelet membrane-coated NPs offer an opportunity to specifically bind the platelet-specific auto-antibodies, thereby preventing them from mediating platelet destruction [28]. Indeed, researchers found that the platelet membrane-coated NPs are highly effective at reducing platelet destruction and preserving normal hemostatic function in vivo. The so called “nanomotors” in platelet membranes were cloaked and used to mediate long term propulsion in blood [169]. The resultant nanorobotic particles still exhibited platelet-like properties, including significant adhesive ability and the potential to bind toxins and pathogens, such as Shiga toxin and *S. aureus*.

In another example, the nanorobots combining proteins from RBCs and platelets were developed, which require no fuel and are ideal for detoxification efforts [170]. The nanorobots readily binded both pathogens and toxins, serving as an optimal hybrid membrane-coated multifunctional therapeutic strategy.

## Model Drugs and Patents Based on Cell Membrane Coating Technology

### Model Drugs Investigated

Table 3 summarizes the model drugs used in the context of cell membrane biomimetics. While the selected drugs have recognized good therapeutic efficacy, they also have some limitations, such as multi-drug resistance (e.g., DOX), poor targeting, or poor water solubility (e.g., PTX and GA). A major focus of cell membrane-related biomimetic drug research is thus unsurprisingly focused on overcoming multidrug resistance, prolonging drug circulation, enhancing drug targeting, and improving therapeutic efficacy. Cell membrane coating allows drugs having similar characteristics to those of the cell membrane. The membrane-associated properties, including extended circulation, immune escape, inflammation targeting, or tumor targeting could directly improve the drug efficacy. Cell membrane coating not only increase the biocompatibility of drugs in vivo but also avoid consequent rapid clearance. The natural targeting strategies, such as platelet or cancer cell membranes, can reduce systemic drug distribution, thus achieving maximal therapeutic targeting and reducing associated side effects. In addition, using appropriate carrier modifications can not only prevent drug leakage, but also achieve controlled drug release.

**Table 3:** A summary of model drugs used in cell membrane coating technology-related research

Drugs	Molecular weight (MW)	Water solubility	Types of enveloped cell membranes	Inner core materials	Drug loading (%)	Encapsulation efficiency (%)	Indication(s)	References
Vinca alkaloid vincristine	824.972	2.27 mg L^−1^	RBC membrane	Solid lipid nanoparticle	2.1	55.72	Glioma	[88]
Gambogic acid	628.762	Insoluble	RBC membrane	PLGA	/	/	Colorectal cancer	[87]
Doxorubicin	543.525	Insoluble	RBC membrane	MSN	39.8	97.6	Breast cancer	[39]
			Platelet membrane	Nanogel	/	/	Breast cancer	[106]
				PLGA	/	/	Breast cancer	[171]
				Liposome	11	/	Breast cancer	[110]
			Monocyte Cell membrane	PLGA	21	/	Breast cancer	[172]
			Cancer cell membrane	Magnetic iron oxide nanoparticle	16.8	/	Hepatoma	[137]
				Hollow silica interlayer (hSiO2)	10	/	Breast cancer	[147]
				Iron oxide	1.8	/	Osteosarcoma	[173]
				Au nanocages	8.1	/	Hepatoma	[40]
				MSN	4.2	/	Prostate cancer	[143]
			Stem cell membrane	PLGA	/	/	Hepatoma	[151]
				Nanogel	15	/	Cervical cancer	[50]
			Hybrid cell membrane (Red blood cell membrane and Melanoma cell membrane)	PLGA	87.7	95.5	Melanoma	[161]
Vancomycin	1449.265	2.25 × 10^−1^ g L^−1^	Bacterial membrane	PLGA	/	36	Staphylococcus aureus bacteremia	[58]
Rapamycin	914.187	9.9 × 10^−5^ mg L^−1^	RBC membrane	PLGA	7.79	/	Atherosclerosis	[83]
			Platelet membrane	PAMAM	/	84.5	Restenosis	[112]
Quercetin	302.238	60 mg L^−1^	Macrophage membrane	Hollow bismuth selenide	/	/	Breast cancer	[132]
Docetaxel	807.89	Insoluble	Platelet membrane	PLGA	4	/	Coronary restenosis	[111]
Paclitaxel	853.918	Insoluble	RBC membrane	Au nanoparticle	4.6	/	Breast cancer	[99]
				PCL	4.1	96.8	Breast cancer	[174]
			Cancer cell membrane	PCL	4	96.02	Breast cancer	[141]
Metformin	129.167	Soluble	Platelet membrane	W_18_O_49_ nanoparticle	2.78	69.83	Burkitt’s lymphoma	[116]
Rifampicin	822.953	1400 mg L^−1^	Bacterial membrane	PLGA	/	/	Staphylococcus aureus bacteremia	[58]
NR2B9C	/	Soluble	RBC membrane	Dextran	/	/	Ischemic stroke	[131]
Glyburide	494.01	Insoluble	Stem cell membrane	PLGA	5	/	Stroke	[175]
Sorafenib	464.825	Insoluble	Cancer cell membrane	Iron oxide	8.6	94.6	Glioma	[176]

Nowadays, cell membrane coating technology is mainly focused on cancer treatment in combination with specific drugs. Owing to the excellent biocompatibility of cell membranes, the model drugs studied are generally insoluble drugs to improve the solubility. Beyond cancer, the damage and repair-associated properties of cell membranes, as in the case of platelets, make them well-suited to be coated on the NPs and treat other diseases such as atherosclerosis.

### Recent Patents

With the development and application of CM-NPs, there have been an increasing number of relevant patents filed over the past decade. Table 4 lists applications for bionic patents for cell membrane coating technologies from year 2009 to 2018.The applications highlight a trend wherein biomimetic drug design is gradually expanding beyond initial RBC membrane efforts to a more diversified array of membrane types. US20130337066A1 described the synthesis of an erythrocyte membrane-camouflaged PLGA NPs designed for long-circulating cargo delivery, fabricating cell-mimicking NPs through a top-down approach. The biomimetic delivery platform may represent an elegant method for personalized medicine whereby the drug delivery nanocarrier is tailored to individual patients with little risk of immunogenicity by using their own RBC membranes as particle coatings. Similarly, a biomimetic nanosystem of activated neutrophil membrane-coated PLGA loaded chemotherapeutic drug NPs has been invented (CN201610803744.6). The biomimetic nanosystem can target circulating cancer cells and achieves strong targeting and high efficiency.

**Table 4:** A list of selected patents and patent applications related to cell membrane coating technology (Data obtained on 09/23/2019)

Patent/application number	Patent title	Cell membrane	Assignee	Filing year	Status
US7901674B2	Aldehyde-fixed platelets with internalized paramagnetic or magnetic nanoparticles	Red blood cell membrane/platelet membrane	The University of North Carolina at Chapel Hill, Chapel Hill, NC (US)	2007	Granted
US20130071329A1	Theranostic delivery systems with modified surfaces	Leukocyte membrane	Board of Regents of the University of Texas System, Austin, TX (US)	2011	Filed
US10117886B2	Hyaluronidase and a low-density foreign patent documents second PEG layer on the surface of therapeutic-encapsulated nanoparticles to enhance nanoparticle diffusion and circulation	Red blood cell membrane	/	2015	Granted
US201702740591A1	Self-antigen displaying nanoparticles targeting auto-reactive immune factors and uses thereof	Red blood cell membrane	The Regents of the University of California, Oakland, CA (US)	2015	Filed
US20170079909A1	Hydrogel toxin-absorbing or binding nanoparticles	Red blood cell membrane	The Regents of the University of California, Oakland, CA (US)	2015	Filed
US20170143830A1	Cellular micromotors and uses thereof	Red blood cell membrane	The Regents of the University of California, Oakland, CA (US)	2016	Filed
US20180140558A1	Detoxification using nanoparticles	Red blood cell membrane	The Regents of the University of California, Oakland, CA (US)	2016	Filed
US20180153821A1	Treating vasculature related diseases or disorders using nanoparticles	Platelet membrane	The Regents of the University of California, Oakland, CA (US)	2016	Filed
US20180169027A1	Treating infection by a platelet-targeting microbe using nanoparticles	Platelet membrane	The Regents of the University of California, Oakland, CA (US)	2016	Filed
US20180200194A1	Decoy nanoparticles to disrupt cancer cell-stromal cell networks	Cancer cell membrane	The John Hopkins University, Baltimore, MD (US)	2016	Filed
US20180085320A1	Modulating antibacterial immunity via bacterial membrane-coated nanoparticles	Bacterial membrane	The Regents of the University of California, Oakland, CA (US)	2016	Filed
EP2714017B1	Membrane encapsulated nanoparticles and method of use	Red blood cell membrane	The Regents of the University of California, Oakland, CA (US)	2012	Granted
WO2016109306Al	Use of nanoparticles coated with red blood cell membranes to enable blood transfusion	Red blood cell membrane	Cellics Therapeutics, Inc., San Diego, CA (US)	2015	Filed
WO2017087897Al	Processes and systems for preparing cellular or viral membranes and nanoparticles	Red blood cell membrane	Arytha Biosciences, LLC, San Diego, CA (US)	2016	Filed
WO2017027760Al	Platelet membrane-coated drug delivery system	Platelet membrane	North Carolina State University, Raleigh, NC (US)	2016	Filed
WO2017120342A1	Cellular or viral membrane coated nanostructure and uses thereof	Beta cell membrane	The Regents of the University of California, Oakland, CA (US)	2017	Filed
CN201610148434.5	Antibody nanoparticles encapsulated in erythrocyte membranes for delivery of antibody drugs and their preparation methods	Red blood cell membrane	East China Normal University, Shanghai, China	2016	Granted
CN201610803744.6	An activated neutrophil membrane coated biomimetic nanoparticles and its preparation method	Neutrophil membrane	Fudan University, Shanghai, China	2016	Filed
CN201710084446.0	Polyester-loaded arsenic trioxide nanoparticles encapsulated by erythrocyte membrane and its preparation method	Red blood cell membrane	Shanghai Jiao Tong University, Shanghai, China	2017	Filed
CN201711173732.0	Preparation and application of biomimetic drug delivery system of cell membrane targeting atherosclerotic lesions	Macrophage membrane	Southwest University, Chongqing, Chian	2017	Filed
CN201711431735.X	Preparation and application of a hollow mesoporous titanium dioxide biomimetic drug complex coated with cancer cell membrane and loaded with autophagy inhibitors	Cancer cell membrane	Zhengzhou University, Zhengzhou, China	2017	Filed
CN201810074719.8	Preparation and application of berberine hydrochloride nanoparticles encapsulated by erythrocyte membrane	Red blood cell membrane	Shanghai Jiao Tong University, Shanghai, China	2018	Filed
CN201811003523.6	An erythrocyte membrane-coated PLGA nanocarrier loaded with anticancer drugs and its preparation and application	Red blood cell membrane	Donghua University, Shanghai, China	2018	Filed
CN201810642330.9	A photosensitive cell membrane biomimetic targeted nanodrug for combined tumor therapy and its preparation	Red blood cell membrane	Jinan University, Guangzhou, China	2018	Filed
CN201811055247.8	Preparation of biomimetic red blood cell membrane nanoparticles with targeting and photothermal integration	Red blood cell membrane	Zhejiang Sci-Tech University, Hangzhou, China	2018	Filed
CN201810140423.1	Biomimetic rapamycin nanoparticles coated with platelet membrane targeting atherosclerotic plaque and its application	Platelet membrane	Zhongshan Hospital Fudan University, Shanghai, China	2018	Filed
CN201810766716.0	Preparation and application of a bio-camouflage targeted nano-delivery system for the treatment of ischemic stroke	Platelet membrane	China Pharmaceutical University, Nanjing, China	2018	Filed
CN201810122851.1	Biomimetic nanoparticles coated with membranes of eukaryotic cells against rotavirus infection and their preparation methods	CaCO_2_ cell membrane/MDCK cell membrane	Institute of Medical Biotechnology, Chinese Academy, Beijing, China	2018	Filed

WO2017027760Al describes a platelet membrane-coated drug delivery system which can sequentially and site-specifically deliver both extracellularly active drugs and intracellularly functional drugs to cancer cells. By taking advantage of the high affinity between platelet membranes and cancer cells, the platelet membrane-coated nanovesicle effectively aggregates on the surface of cancer cells and can thereby promote the interaction of extracellularly active drugs. After endocytosis, platelet membrane-coated nanovesicles can be degraded by acidity in the lyso-endosome, accompanied by the release and further accumulation of encapsulated intracellularly functional drugs. Jinan University has proposed a method for preparing human erythrocyte membrane-coated NPs for drug combination photodynamic therapy of tumors in its patent. This delivery platform can not only realize the high-efficiency loading of drugs, but also can effectively prolong their circulation time in vivo, so as to achieve accurate and continuous drug delivery in the tumor.

These patents clearly demonstrate that an effective cargo delivery system can be constructed by using cell membrane coating technology, offering a potential novel platform for disease treatment. With the development of this technology, the application of cell membranes has expanded from blood cells to immune cells, tumor cells, and other cell types. Based upon patent applications from the last 2 years, we can see that in addition to increases in cell membrane types utilized, the relatively mature cell membrane delivery systems (e.g., RBCM-NPs) are also progressing in a more efficient and intelligent direction.

## Conclusion and Future Perspective

The unique properties of natural cell membranes, including their ability to facilitate extended circulation time, immune escape, adhesion, and homologous targeting, have led to the novel application of membrane coating technology in the context of nanomedicine. Cell membrane used include red blood cell membranes, platelet membranes, white cell membranes (macrophage, neutrophils, and T cell membranes), cancer cell membranes, stem cell membranes, beta cell membranes, fibroblast cell membranes, and their hybrid membranes. The CM-NPs have been shown to be effective for drug delivery, phototherapy, immune modulation, and detoxification.

Extrusion and ultrasound production are the main methods to prepare NPs coated with cell membranes. In recent years, microfluidic electroporation has also been proposed as a means of preparing more stable and uniform CM-NPs. However, the clinical preparation of such CM-NPs remains challenging. Because this technology relies upon a simple top-down preparation method, the requirements for nuclear materials are not stringent, thus providing ample opportunity for the biomimetic utilization of a wide range of materials and drug dosage forms. Polymers, liposomes, silica, iron oxide NPs, and metal materials can all be encapsulated in cell membranes. New two-dimensional materials (e.g., BP) and some drug formulations (e.g., micelles, nanogel, and nanocrystals) have also been combined with cell membrane coating technologies. The diverse array of combinations thus greatly expands the scope of cell membrane coating technologies.

The membrane coating technology is mainly used for cancer treatment, with studies in breast cancer, liver cancer, colon cancer, head and neck squamous cell tumors, melanoma, and other tumor models, and achieved good experimental therapeutic efficacy in vivo and in vitro. As foreign substances interact with cell membranes, nanosponges have been successfully developed that take advantage of this property to achieve effective detoxification outcomes. The diverse array of available cell membrane types offers ample opportunity to treat a diverse range of diseases. In particular, platelet membranes have the potential to be utilized to treat atherosclerosis and restenosis. Immune cell membranes, in contrast, offer an opportunity to treat immunological diseases. The emergence of hybrid cell membranes allows for the blending of the characteristics of various cell membrane types, optimizing their functionality. This hybrid strategy has been developed extensively over the past 2 years. We believe that the development of hybrid cell membrane coating technologies will remain a major area of active research in the near future.

However, there are still some limitations associated with cell membrane coating technologies. In order to develop multi-functional intelligent CM-NPs, certain membrane modifications will inevitably be required, potentially inducing undesirable side effects. Excessive use of immune CM-NPs may induce or aggravate inflammation through interactions with the immune system, thereby leading to pathological mediator release.

Herein, we additionally surveyed common model drugs and recent CM-NP-related patents that have been filed in the past decade. The CM-NPs have the potential to increase chemotherapeutic drug biocompatibility while decreasing the incidence of associated side effects. In addition, relatively mature cell membrane delivery systems (e.g., blood CM-NPs, immune CM-NPs, and cancer CM-NPs) are being developed in a more intelligent and effective manner. Although the technology of cell membrane coating has not yet achieved full clinical implementation, its clear advantages and the abundant sources of cell membrane offer a solid foundation for its industrial production and implementation in individual precision medicine approaches. We believe that in the near future, the research and development of CM-NPs will yield invaluable contributions to human health.
